# Enhanced expression of the myogenic factor Myocyte enhancer factor-2 in imaginal disc myoblasts activates a partial, but incomplete, muscle development program

**DOI:** 10.1016/j.ydbio.2024.08.004

**Published:** 2024-08-05

**Authors:** Elizabeth M. Trujillo, Samuel R. Lee, Antonio Aguayo, Tylee C. Torosian, Richard M. Cripps

**Affiliations:** Department of Biology, San Diego State University, San Diego, CA, 92182, USA

**Keywords:** Drosophila, Myocyte enhancer factor-2, MEF2, Myosin, Myogenesis, Myoblast

## Abstract

The Myocyte enhancer factor-2 (MEF2) transcription factor plays a vital role in orchestrating muscle differentiation. While MEF2 cannot effectively induce myogenesis in naïve cells, it can potently accelerate myogenesis in mesodermal cells. This includes in *Drosophila melanogaster* imaginal disc myoblasts, where triggering premature muscle gene expression in these adult muscle progenitors has become a paradigm for understanding the regulation of the myogenic program. Here, we investigated the global consequences of MEF2 overexpression in the imaginal wing disc myoblasts, by combining RNA-sequencing with RT-qPCR and immunofluorescence. We observed the formation of sarcomere-like structures that contained both muscle and cytoplasmic myosin, and significant upregulation of muscle gene expression, especially genes essential for myofibril formation and function. These transcripts were functional since numerous myofibrillar proteins were detected in discs using immunofluorescence. Interestingly, muscle genes whose expression is restricted to the adult stages were not activated in these adult myoblasts. These studies confirm a broad activation of the myogenic program in response to MEF2 expression and suggest that additional regulatory factors are required for promoting the adult muscle-specific program. Our findings contribute to understanding the regulatory mechanisms governing muscle development and highlight the multifaceted role of MEF2 in orchestrating this intricate process.

## 1. Introduction

Skeletal muscles are composed of myofibers organized into bundles, called fascicles. These myofibers are made up of myofibrils which, in turn, are structured from repeating contractile units known as sarcomeres that extend from Z-disc to Z-disc. Sarcomeres consist of overlapping arrays of thin actin and thick myosin filaments which gives skeletal muscles their characteristic striated appearance. As the sarcomeres within the myofibers shorten, the myofibers contract, leading to movement and locomotion (Alberts B et al., 2002; Huxley and Niedergerke, 1954; Hill AV, 1938). The proper functioning of skeletal muscles is vital to performing essential roles in respiration, stability, and movement. Many developmental deficiencies can disrupt normal physiological processes leading to an array of muscle dystrophies and/or myopathies (Thomas, 2013).

In mammals, the development of muscles involves a complex interplay of many regulatory genes, that control events from the fusion of myoblasts, through the formation of premyofibrils, to the maturation of myotubes into contractile fibers (reviewed in Lehka and Rędowicz, 2020; Sanger et al., 2002). The complex process of myofibrillogenesis is activated by transcription factors including Myocyte enhancer factor 2 (MEF2) and the myogenic basic helix-loop-helix proteins MyoD, myf-5, Myogenin, and MRF-4 (reviewed in Hernández-Hernández et al., 2017). Prominent among these genes is MEF2, whose central role in muscle development is to trigger myoblast fusion (reviewed by Haralalka and Abmayr, 2010; Bour et al., 1995; Brunetti et al., 2015; Bryantsev et al., 2012a) and muscle differentiation (Lilly et al., 1995; Bour et al., 1995; Lin et al., 1997). MEF2 additionally regulates cardiac development and function (Desjardins and Naya, 2016), and neuronal development (reviewed in Dietrich, 2013), and has a direct involvement in human diseases such as cancers (reviewed in Chen et al., 2017). In *Drosophila melanogaster*, MEF2 regulates cardiac tube development (reviewed in Bileckyj et al., 2023), pacemaker neurons (Blanchard et al., 2010), and immune responses (Clark et al., 2013). Moreover, MEF2 directly regulates the expression of numerous target genes that are essential for muscle development, including *Act57B*, *Mlc1*, *Mlc2*, and *Mhc* (Kelly et al., 2002; Elgar et al., 2008). However, despite an extensive list of known MEF2 target genes, MEF2 alone cannot convert naïve cells into muscles, either in mammals or *Drosophila* (Molkentin et al., 1995; Abmayr et al., 1995). By contrast, when expressed in a mesodermal environment, MEF2 can potentiate muscle gene expression in myoblasts. For example, in embryos, MEF2 plays a crucial role in initiating a genetic cascade that starts in the committed mesoderm, progresses to the somatic mesoderm, and ultimately results in the formation of skeletal muscle cells (Lilly et al., 1995; Bour et al., 1995).

Several previous studies have shown that differentiation can also be triggered in the wing imaginal disc myoblasts. These undifferentiated mesodermal cells are set aside in the embryo, proliferate during the larval stage, and differentiate during metamorphosis to ultimately give rise to the adult wing and thoracic muscles (Bate et al., 1991; Fernandes et al., 1991). The *Drosophila* thoracic muscular system is largely composed of fibrillar indirect flight muscles (IFMs) responsible for powering the wing stroke during flight (Bernstein et al., 1993; Spletter et al., 2018), and the tergal depressor of the trochanter muscle (TDT, or jump muscle) responsible for jumping (Nachtigall and Wilson, 1967; Zumstein et al., 2004; Jaramillo et al., 2009). Premature muscle differentiation can be triggered in wing disc myoblasts either by increasing expression of *Mef2* (Lovato et al., 2005; Soler and Taylor, 2009; Boukhatmi and Bray, 2018) or by knocking down expression of the *Zfh1* gene (Boukhatmi and Bray, 2018), that encodes a myogenic repressor (Postigo et al., 1999). However, to date, studies of muscle differentiation in the wing discs have relied predominantly upon assessing the accumulation of candidate myofibrillar proteins (Myosin heavy-chain and Tropomyosin) or F-actin; the extent to which a broader myogenic program is initiated has not been investigated.

Here, we carried out cell biological and transcriptomic analyses to identify the breadth of the myogenic program that MEF2 can trigger in wing disc adult myoblasts. We found that MEF2-induced myofibrillar proteins accumulated both into striated and non-striated structures. Some of these structures had characteristics of pre-myofibrils found in early differentiating muscles, but there was no extensive fusion of myoblasts into syncytia. At the transcriptomic level, there were over 147 up-regulated genes, mostly associated with muscle structure and function, and the increased expression of several of these was confirmed through immunofluorescence and quantitative RT-PCR (qPCR). Since the adult muscles express novel muscle proteins or unique isoforms of canonical muscle proteins, we also determined if the transcriptional profile from these adult myoblasts more closely resembled embryonic/larval or pupal/adult muscles. We found a strong bias towards the expression of embryonic/larval muscle protein genes, indicating that factors in addition to MEF2 are required for the proper formation of the more complex adult muscles.

## 2. Materials and Methods

### 2.1. Drosophila stocks and crosses

All crosses were maintained in vials or bottles containing Jazz Mix medium (Genesee Scientific) at 25 °C, where they were allowed to lay eggs, and then transferred to 29 °C incubators after three days to maximize Gal4-induced expression. The following fly stocks were used: *1151-Gal4; fln-LacZ* (myoblast specific driver, Bryantsev et al., 2012b); *w UAS-Mef2 [26A]* (overexpression of *Mef2*, Lovato et al., 2005); and *w^1118^* (control). To trigger premature *Mef2* expression in the imaginal discs, we crossed *1151-Gal4; fln-lacZ* to *w UAS-Mef2 [26A]*. For controls we crossed *1151-Gal4; fln-lacZ* to *w^1118^*. Since both *1151-Gal4* and *UAS-Mef2* are on the X chromosome, we analyzed solely female offspring for these experiments.

### 2.2. RNA extraction

Wing imaginal discs were dissected from wandering third instar female larvae and then stored in Qiagen Buffer RLT, at −80 °C, until a total of 60 wing discs were collected, per replicate. Then, total RNA was extracted from each replicate using RNeasy Micro Kit (#74104; Qiagen, USA). Using a NanoDrop 2000 (Thermo Fisher Scientific, USA) the average yield extracted from control replicates was 1919.80 ng/μl (20 μl total extraction volume) and the average yield extracted from mutant replicates was 2387.97 ng/μl (20 μl total extraction volume). The quality of the RNA was determined by UC San Diego IGM Genomics Center using RNA ScreenTape^®^ analysis (Agilent, USA). The average RIN^e^ for control and experimental samples was 10.0 (Fig. S1).

### 2.3. Transcriptomic analysis

RNA-sequencing (RNA-seq) was conducted using Illumina NovaSeq 6000. Three single-end libraries were made from both control and experimental samples. No trimming was used. The modern aligner HISAT2 was used to map reads to Drosophila reference genome dm6 (*D. melanogaster* Aug. 2014 BDGP Release 6 + ISO1 MT/dm6) (Kim et al., 2019). Quality control was completed using the fastqcr R package. Next, to quantify the expression level of transcripts, the alignment files were used as inputs for featurecounts (Liao et al., 2014). Then, DESeq2 was used to identify significantly expressed genes between the control and experimental samples using these counts (Love et al., 2014). P < 0.05 was set as the threshold for significantly differentially expressed genes. Gene ontology (GO) analysis was completed with ClueGO, a CytoScape plug-in (Bindea et al., 2009). The raw and analyzed data were deposited in the Mendeley Data database under https://doi.org/10.17632/777hyfy6h5.1.

### 2.4. Quantitative reverse transcription-polymerase chain reaction (RT-PCR)

1 μg of total RNA from imaginal wing disc replicates was used to synthesize cDNA with iScript Adv cDNA kit for qPCR (#1725038; Bio-Rad) using C1000 Touch Thermal Cycler (Bio-Rad, USA). Then, SsoAdvanced Universal SYBR Green Supermix (#1725270; Bio-Rad, USA) was used for quantitative PCR (qPCR) using the CFX96 Real-Time System (Bio-Rad, USA). Each sample was run in triplicate and the transcripts were analyzed by 2^−ΔΔ*CT*^ method to calculate the relative gene expression. The threshold for significantly differentially expressed genes was set at p-value below 0.05. The mean ± standard error of the mean was used for all graphs. The relative quantification was standardized to *Act5C* RNA levels. Primers used were as follows.

Act5C-Forward: 5′-GCAAGTACTCTGTCTGGATC – 3′

Act5C-Reverse: 5′-CCAGCAGAATCAAGACCATCC – 3′

Act57B Forward: 5′- TGCAGTTGCCTAGCACCA – 3′

Act57B Reverse: 5′- GACCCCATCCGCTCTATC – 3′

Act79B Forward: 5′- TGTCTCCAGCGTAAGACATCC – 3′

Act79B Reverse: 5′- TTCCGGTCTTTTCTCGTCTC – 3′

Act88F Forward: 5′- AGCTCTTCAAAGGCAGCAAC – 3′

Act88F Reverse: 5′- ATTGTTGTGCGATGGGTTC – 3′

TnI Forward: 5′- GAAGGTCTCCAAATACGAAA – 3′

TnI Reverse: 5′- GCTGTTGTTGTTTATTGACTTC – 3′

MHC Forward: 5′- AGAAGGCTGAGGAACTGC – 3′

MHC Reverse: 5′- GTTCAAGTTGCGGATCTG – 3′

To assess patterns of alternative RNA splicing, diluted cDNA was used for PCR using OneTaq 2X Master Mix with Standard Buffer (New England Biolabs). The following primers were used.

MHC Forward: 5′- ATGGTGAGCAGAGGAGGCA – 3′

MHC Reverse: 5′- CGTTTTCAGGAGCAAGGTCG – 3′

Mlc1 Forward: 5′- GAGAGCTTGGATGACGAGCA – 3′

Mlc1 Reverse: 5′- GGTTCAGCCAAAAGCAAAGC – 3′

Sls Forward: 5′- CGCGCAGTATGTGCAAAAT – 3′

Sls Reverse: 5′- AAACCGTTCCACGAAAAGTG – 3′

The PCRs were run for 20–35 cycles and the final products were resolved in a 2% agarose gel.

### 2.5. Immunofluorescent analysis

Wing imaginal discs from wandering third instar female larvae were dissected in 1X phosphate buffer saline (PBS) and fixed in 3.7% formaldehyde for 30 min at 4 °C, followed by immunolabeling. All primary antibodies were incubated with the samples overnight at 4 °C and secondary antibodies were incubated for 2 h at 22 °C. Samples were mounted in mounting media. Antibodies recognizing Myofilin (Mf), Myosin alkali light chain 1 (Mlc1), and Troponin T (TnT) did not show protein expression in the experimental discs (Table 2). However, we believe that these antibodies are not functional in our hands because cryosections of *w^1118^* adult thoracic muscles did not show any specific staining (data not shown). See the reagent table for details of the antibodies used.

### 2.6. Imaging and statistical analysis

Immunostained preparations were mounted in mounting media and imaged on an Olympus FluoView 3000 confocal microscope. Images were processed using Olympus Life Science software and assembled in Adobe Photoshop (2024) (version 25.0). All experimental and confocal images underwent equal changes in brightness and contrast.

To assess the extent of muscle protein accumulation relative to the total myoblast area, an area was drawn around MHC, F-actin, and MEF2 positive staining to calculate the fraction of the myoblast area occupied by MHC or F-actin. To calculate this fraction, the area occupied by F-actin or MHC was divided by the area occupied by myoblasts (MEF2 staining).

To estimate the size of the myoblast pool, a border was also drawn around myoblasts, stained with anti-Cut, anti-MEF2 or anti-Zfh1 antibodies, and the area within that border was calculated using ImageJ. Immunopositive areas were defined as those that showed nuclear accumulation of the myoblast transcription factors. The error bars on all the graphs are standard error of the mean. Student’s two-tailed *t*-test was used to calculate the p-value.

Data for qPCR was analyzed using parametric, two-tailed, unpaired Welch’s *t*-test, using Microsoft^R^ Excel (version 16.77.1). GraphPad Prism software (Version 10.0.3) was used to generate all pie charts and bar graphs. The error bars are presented as the standard error of the mean. P < 0.05 was set as the threshold for significance and marked by asterisks.

## 3. Results

### 3.1. Phenotypic analysis of MEF2-induced myogenesis in wing discs

We first studied the types of muscle-like structures that were formed in the wing imaginal discs of late third-instar larvae following *Mef2* over-expression. We used the *1151-Gal4* driver line, which is active in the adult myoblasts (Anant et al., 1998), and crossed females to either *w^1118^* control males or to *UAS-Mef2* males (Lovato et al., 2005). Since both *1151-Gal4* and *UAS-Mef2* are X-linked, we analyzed only female offspring of control and experimental crosses. We assessed *Mef2* expression levels using RT-PCR (qPCR), and found that there was a 1.901 log fold change and a p-value of 0.02 (p-value <0.05 being significant) among *1151>Mef2* discs compared to controls (Fig. S2), confirming the over-expression of *Mef2* in these samples.

**Fig. 1.**
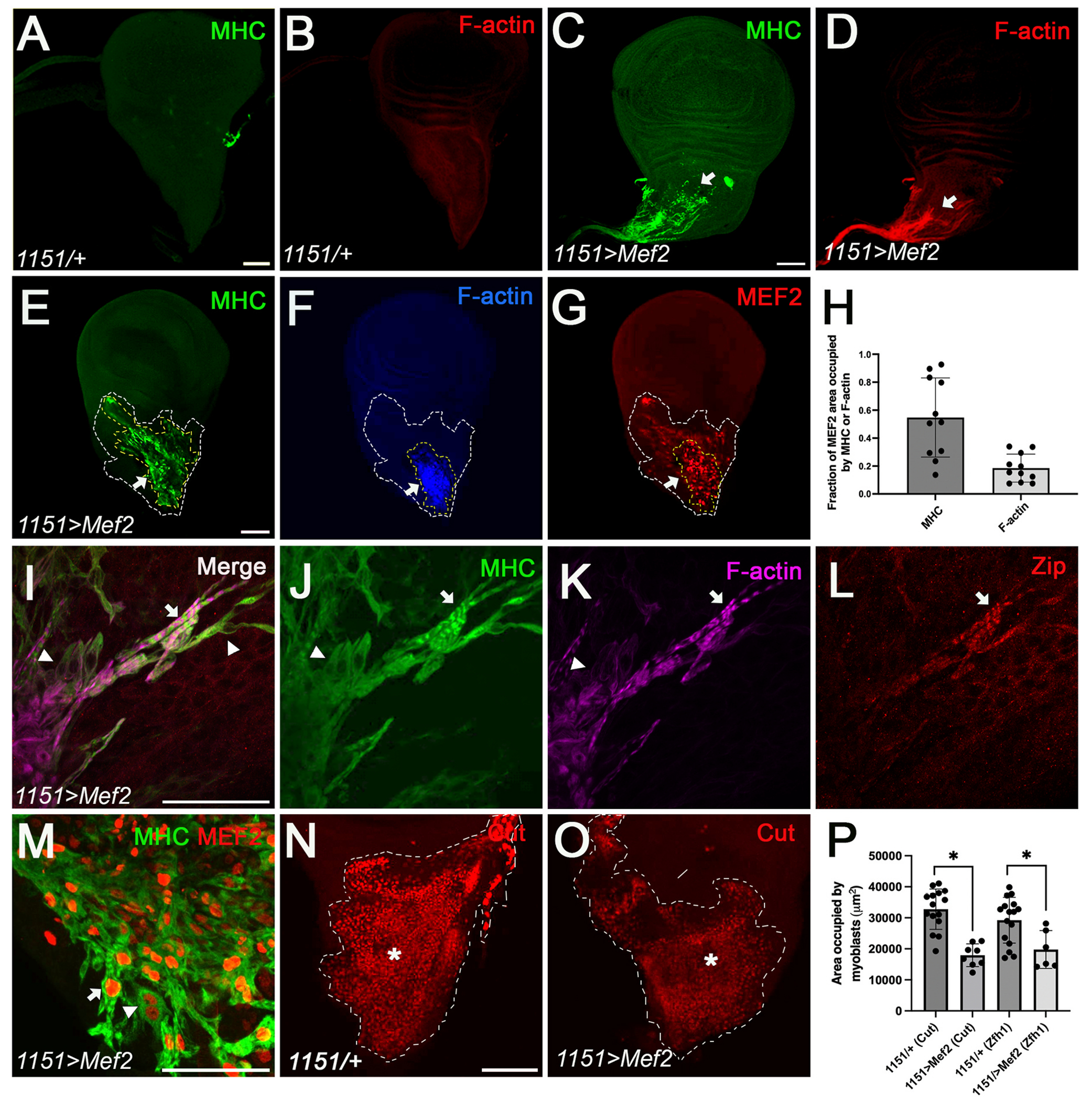
Phenotypic analysis of MEF2-induced myogenesis in wing discs. (A–D) Control (*1151*/+) wing imaginal discs of late third-instar larvae upon *Mef2* overexpression show no expression of myosin heavy chain (anti-MHC, green) and F-actin (skeletal muscles; red) while experimental discs (*1151*>*Mef2*) show expression of both (arrow). Control n = 20. Experimental n = 11. Scale bar: 50 μm. (E–F) MHC and F-actin expression is more prominent at the proximal tip of *1151*>*Mef2* discs (arrow). Wing disc area occupied by MHC (yellow dashed line), F-actin (yellow dashed line), and myoblasts (white dashed line). Scale bar: 50 μm. (G) The strongest MEF2 accumulation is located where F-actin is expressed (yellow dashed line; arrow). Wing disc area occupied myoblasts (white dashed line). Scale bar: 50 μm. (H) MHC and F-actin occupied 54.74% (n = 11) and 18.51% (n = 11), respectively, of the area occupied by myoblasts. (I–L) *1151*>*Mef2* discs were stained with MHC, F-actin, and non-muscle myosin heavy-chain, Zipper (Zip). MHC and F-actin expression were found in both striated (arrow) and non-striated structures (arrowhead), while Zip expression was the strongest in the striated structures (arrow). Scale bar: 50 μm. (M) *1151*>*Mef2* discs stained for MHC and MEF2 showed both striated-like mononucleate (arrow) and non-striated bi-nucleate (arrowhead) cells. Scale bar: 50 μm. (N–O) Control (*1151*/+) and experimental (*1151*>*Mef2*) discs stained with Cut (myoblast, red, white dashed line) Scale bar: 50 μm. (P) Quantification of the area occupied by myoblasts revealed a significant decrease in Cut-positive myoblast pool by approximately 45.98% in the experimental discs (n = 9) compared to the controls (n = 16) (p = 0.0019); and a significant decrease in Zfh1-positive myoblasts by 32.32% in the experimental discs (n=6) compared to the controls (n=16) (p=0.01143). The error bars on all the graphs were calculated by the standard error of the mean. All p-values <0.05 were deemed significant and calculated by two-tailed student’s t-test.

While we never observed accumulation of Myosin heavy-chain (MHC) nor F-actin in control wing discs (Fig. 1A and B; n = 20), 100% of experimental discs showed evidence of muscle differentiation (Fig. 1C and D; n = 11). Within each disc, however, not all myoblasts showed evidence of differentiation: MHC and F-actin on average occupied 54.74% (n = 11) and 18.51% (n = 11), respectively, of the total area occupied by myoblasts as defined by MEF2 staining (Fig. 1E–H). This was always most prominent at the proximal tip of the disc but frequently spread across the field of myoblasts. In addition, the regions that showed the most prominent differentiation by F-actin staining also showed the strongest MEF2 accumulation compared to adjacent myoblasts (Fig. 1G), suggesting that once the myogenic program is initiated, *Mef2* expression can be further up-regulated.

Next, we analyzed the myofibril-like structures that were formed in experimental discs. To achieve this, we performed immunofluorescent staining of wing discs using antibodies that recognized either muscle Myosin heavy-chain (MHC), or the non-muscle myosin heavy-chain, Zipper (Zip). We observed varying degrees of myofibril formation, where both striated myofibril-like structures and non-striated structures formed on the same disc (Fig. 1I). MHC and F-actin accumulation were found in both striated and non-striated structures (Fig. 1J and K), whereas Zip accumulation was the strongest in the striated structures (Fig. 1L). Zip is involved in myofibril assembly, specifically myosin II filament assembly, localizing to the M-line in mature larval muscles (Bloor and Kiehart, 2001; Liu et al., 2008).

Since *Mef2* is required for myoblast fusion (Bour et al., 1995; Ranganayakulu et al., 1995) we also determined if there was evidence for muscle syncytia being formed. To assess this, we conducted immunofluorescent staining on experimental discs to detect the accumulation of MHC and MEF2. In regions where myogenic structures were not overlapping, we observed striated-like and non-striated-like structures (Fig. 1M). However, it was challenging to definitively confirm the predominance of multinucleate or mononucleate cells because many of these structures overlap making it hard to see the individual cells, although in a few cases, we did observe what appeared to be bi-nucleate cells (Fig. 1M, arrowhead). In the next section, we assess the expression levels of myoblast fusion genes to gain a more comprehensive understanding of the potential formation of syncytia.

Finally, given that numerous myoblasts were beginning to differentiate, we hypothesized that this would impact the myoblast pool. To test this, we stained control and experimental discs with anti-Cut to measure the wing disc area occupied by myoblasts (Fig. 1N–O). Cut encodes a homeoprotein that functions as a transcriptional factor in different cells, such as the myoblasts of imaginal wing discs. Quantification revealed that the Cut-positive myoblast area was reduced by 45.98% in the experimental discs (n = 9) compared to the controls (n = 16) (p = 0.0019) (Fig. 1P). To add robustness to these data, an antibody against Zfh1 was also used to stain myoblasts (Fig. S3). We observed the myoblast area was reduced by 32.32% in the experimental discs (n = 6) compared to the controls (n = 16) (p = 0.01143) (Fig. 1P). We conclude that myoblast number is consistently and reproducibly reduced in the experimental discs, perhaps through the initiation of differentiation causing a reduction in myoblast proliferation.

### 3.2. Transcriptomic analysis of wing discs with elevated Mef2 expression

To assess the breadth of the myogenic program induced by MEF2, we conducted a transcriptomic analysis of whole wing discs isolated from control *1151*/+ and experimental *1151>Mef2* female late third instar larvae. RNA-sequencing (RNA-seq) was conducted using Illumina NovaSeq 6000. Three single-end libraries were made from both control and experimental samples. Since paired-end data was not used, no trimming was required. Illumina reads used for differential gene expression in RNA-seq analysis are no longer required to undergo trimming when using modern aligners, such as HISAT2, which was used in this analysis (Williams et al., 2016). HISAT2 was used to map reads to the Drosophila reference genome dm6 (*D. melanogaster* Aug. 2014 BDGP Release 6 + ISO1 MT/dm6) (Kim et al., 2019). To ensure the Illumina data was acceptable, it was subjected to quality control using the fastqcr R package, and all categories received a passing score except for “per base sequence content” and “per sequence GC content” (Figs. S4–S12 and Table S1) (Wingett and Andrews, 2018; Andrews, 2023).

The “per base sequence content” always returns as a Fail score because the first 10–12 bases are a result of hexamer priming that occurs during RNA-seq library preparation so that reverse transcription can occur (Wang et al., 2009). Moreover, a Fail in “per sequence GC content” is usually due to overrepresented sequences, which was not observed here. GC content may affect differential expression results and downstream analysis such as Gene Ontology (Risso et al., 2011), so measuring %GC for all replicates was performed (Table 1). Our results showed that %GC content was between 47% and 50% in all samples, consistent with the GC-content range observed in *Drosophila* genomes (Herold et al., 2008). In addition, the mapping rate was 95%. Together, our results indicate high-quality sequencing data and successful alignment to the *Drosophila* genome.

**Fig. 2.**
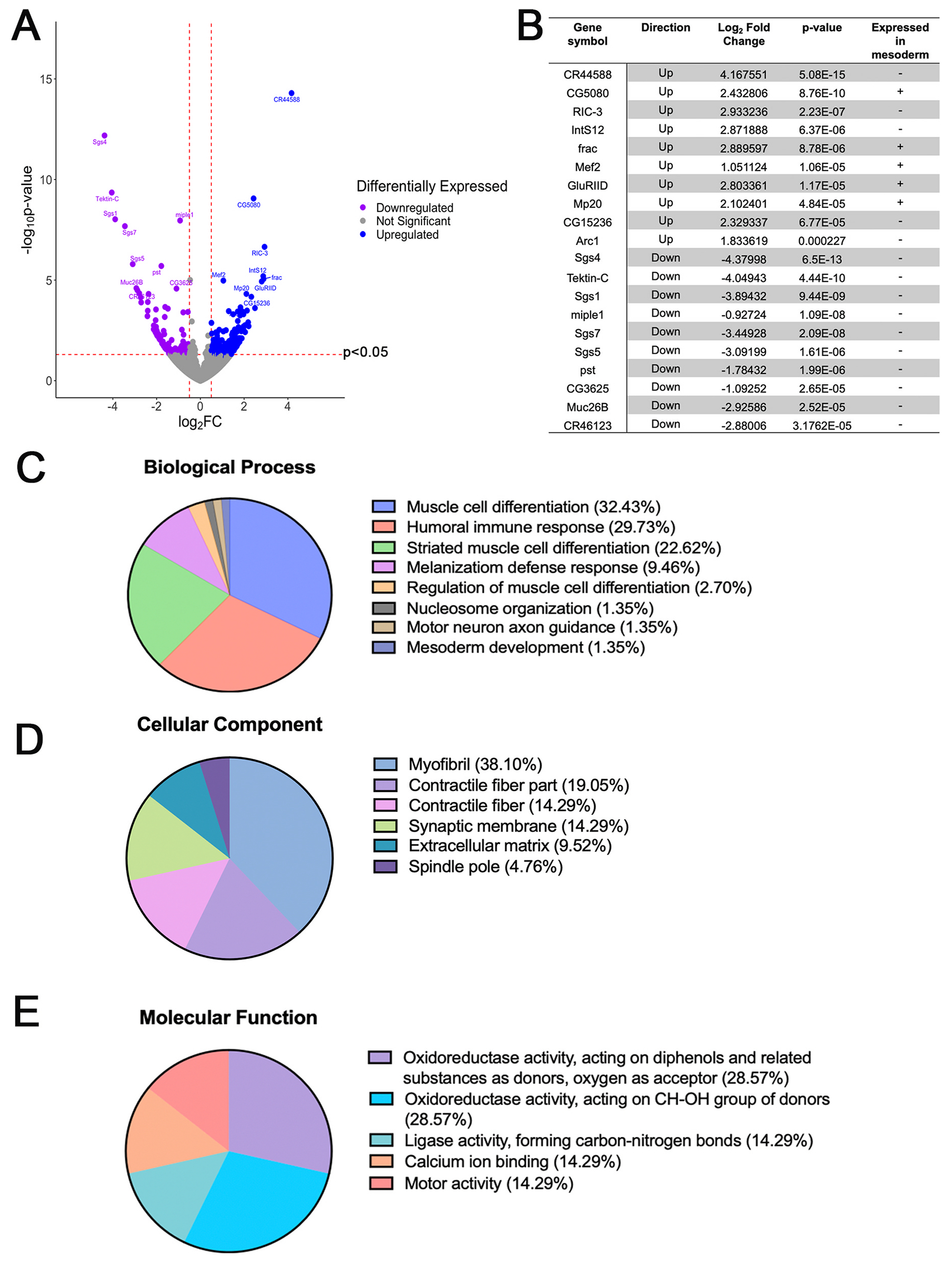
Analysis of differentially expressed genes upon *Mef2* over-expression. Differentially expressed genes (DEGs) were determined using DESeq2 as described in Methods. (A) Volcano plot of DEGs. Log_2_ fold change > or <0.5 plotted against p-value <0.05 was deemed significant. Significant downregulated genes are in purple, significant up-regulated genes are in blue, and non-significant genes are in gray. (B) Table of top 10 up-regulated and downregulated DEGs and their corresponding direction, log_2_ fold change, and p-value. For each gene, a positive sign (+) indicates expression in the mesoderm while a negative sign (−) indicates no currently available data suggesting that the gene is expressed specifically in the mesoderm. (C–E) Pie chart of the Gene Ontology (GO) enrichment analysis of DEGs using ClueGO, a CytoScape plug-in, to analyze the biological processes, cellular components, and molecular functions that were up-regulated. Many differentially regulated genes in Biological Processes relate to muscle cell differentiation (32.43%), while in Cellular Component, the majority are myofibril genes (38.10%), and in Molecular Function, they are genes related to oxidoreductase activity (oxygen as acceptor; 28.57%).

Next, to quantify the expression level of transcripts, the alignment files were used as input for featurecounts (Liao et al., 2014). Then, DESeq2 was used to identify significantly expressed genes between the control and experimental samples using these counts (Love et al., 2014). To visualize downregulated and up-regulated genes, a volcano plot was generated (Fig. 2A). We established significance thresholds at p-value<0.05 and a fold change cutoff of Log_2_FC > 0.5 and Log_2_FC < −0.5, up-regulated and downregulated, respectively, to enable the detection of smaller yet biologically relevant fold changes. This was necessary because the isolated wing discs contain significant amounts of other wing disc tissue in addition to the myoblasts, therefore we were concerned that changes in expression in the myoblasts might be masked by transcripts for the other tissues present. A table listing the top ten significantly up-regulated and downregulated genes was created, showing corresponding p-values, Log_2_ fold changes, and whether they are expressed in the mesoderm, based upon annotations at FlyBase.org (Gramates et al., 2022) (Fig. 2B). From this list, we found that mesoderm-expressed genes are over-represented in the up-regulated fraction.

Next, the differentially expressed genes were used for Gene Ontology (GO) enrichment analysis by means of ClueGO, a CytoScape plug-in, to analyze the biological processes, cellular components, and molecular functions of the 147 genes that were up-regulated in imaginal wing discs with *Mef2* over-expression (Bindea et al., 2009). Analyzing these data showed 32.43% of differentially expressed genes from the Biological Process category are involved in muscle cell differentiation, 38.10% from the Cellular Component category are myofibril genes, and 28.57% from the Molecular Function category have oxidoreductase activity (oxygen as acceptor) (Fig. 2C–E). These data suggest that as the myoblasts undergo differentiation, genes encoding structural proteins are expressed to ensure the proper development and function of myofibrils. In addition, oxygen, which serves as the last electron acceptor in the electron transport chain, enables the cells to generate adenosine triphosphate (ATP) for energy during muscle cell differentiation. Overall, muscle-related and energy-producing GO terms were associated with differentially expressed genes.

For down-regulated genes, although a total of 110 genes were significantly downregulated at Log_2_FC < −0.5, ClueGO only returned the biological process category, showing 80% of the down-regulated genes as “response to ketone” and 20% as “germ cell migration”.

**Fig. 3.**
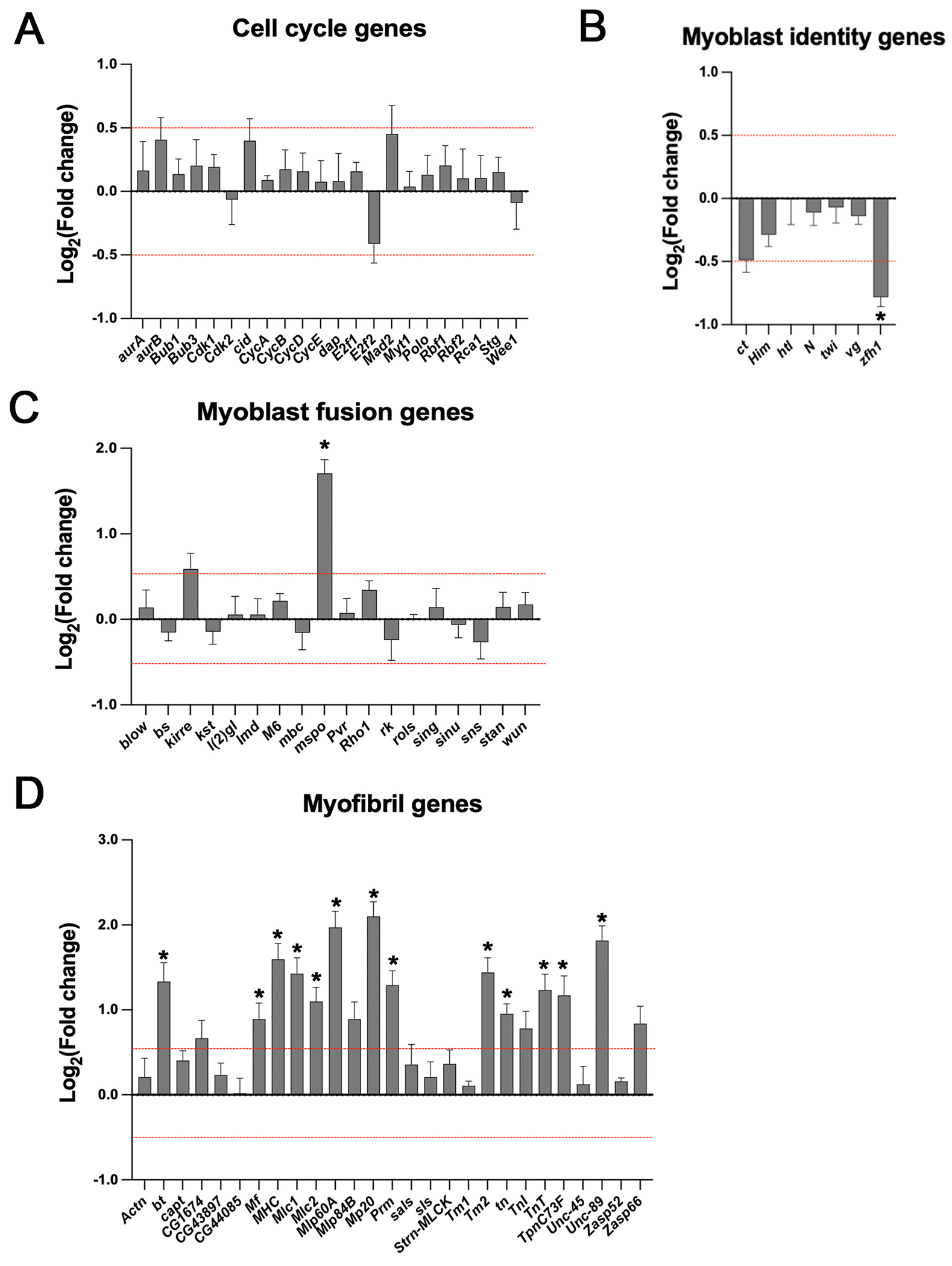
Expression changes for genes associated with cell cycle regulation, myoblast identity, myoblast fusion, and myofibril assembly or function. (A–D) Significant differentially expressed genes (DEGs) in wing imaginal discs of late third-instar female larvae upon *Mef2* over-expression are indicated with an asterisk. Log_2_ fold change greater than or less than 0.5 plotted p-value <0.05 was deemed significant (red dotted line). All graphs are Mean ± Standard Error (Student’s two-tailed *t*-test). No significant DEGs were observed for the cell cycle genes category, while the myoblast identity genes category has one significantly down-regulated gene (*Zfh1*) and the myoblast fusion genes category has one significant upregulated gene (*mspo*). The myofibril gene category revealed 12 significant up-regulated genes, accounting for 48% of the genes in this category.

To gain more specific insight into the differentially expressed genes, we mined the RNA-seq data for known genes associated with myoblast identity, cell cycle regulation, myoblast fusion, and myofibril assembly or function. We found that 0% of the cell cycle genes were differentially expressed (Fig. 3A). Note that our data are from whole discs rather than purified myoblasts, and it is expected that cell cycle genes are broadly expressed in the growing wing discs. Therefore, changes in the expression of these genes solely in the myoblasts may be below the levels of detection.

When we mined the RNA-seq data for myoblast identity genes, we found that one was significantly down-regulated, *Zfh1* (Fig. 3B). The down-regulation of *Zfh1* is consistent with our findings of a reduction of Zfh1-positive myoblasts (Fig. 1P and Fig. S3) (Boukhatmi and Bray, 2018). We also observed a reduction in the expression level of Cut (Fig. 3B) consistent with the reduction in the field of Cut-positive myoblasts (Fig. 1P). However, this reduction was not significant, which may be due to the expression of Cut in other wing disc tissues, including the wing margin, that would not be affected by manipulation of *Mef2* expression in the myoblasts (Blochlinger et al., 1988, 1990; Ludlow et al., 1996).

We also determined if genes associated with myoblast fusion were up-regulated in the experimental samples. We observed that, of 18 genes analyzed, only *mspo* was significantly up-regulated (Fig. 3C). These data are consistent with the minor proportion of syncytial muscle cells that we observed (see Fig. 1). When we analyzed experimental discs in white pre-pupa, we observed striated-like structures and multinucleation (Fig. S13; white bracket). These data suggest that additional myoblast fusion genes are being activated during this later stage of development resulting in more evidence of myoblast fusion, although we have not tested this experimentally.

Finally, we assessed the expression levels of known sarcomeric protein genes. We observed that all genes studied showed up-regulation of expression in response to MEF2, and 48% of these genes showed significantly up-regulated expression (Fig. 3D). Overall, our RNA-seq and GO analysis highlight the potential role of MEF2 in causing muscle differentiation and myofibril formation, although it appears that not all myogenic processes, such as myoblast fusion, are significantly impacted.

### 3.3. Immunofluorescence analysis of wing discs with elevated Mef2 expression

**Fig. 4.**
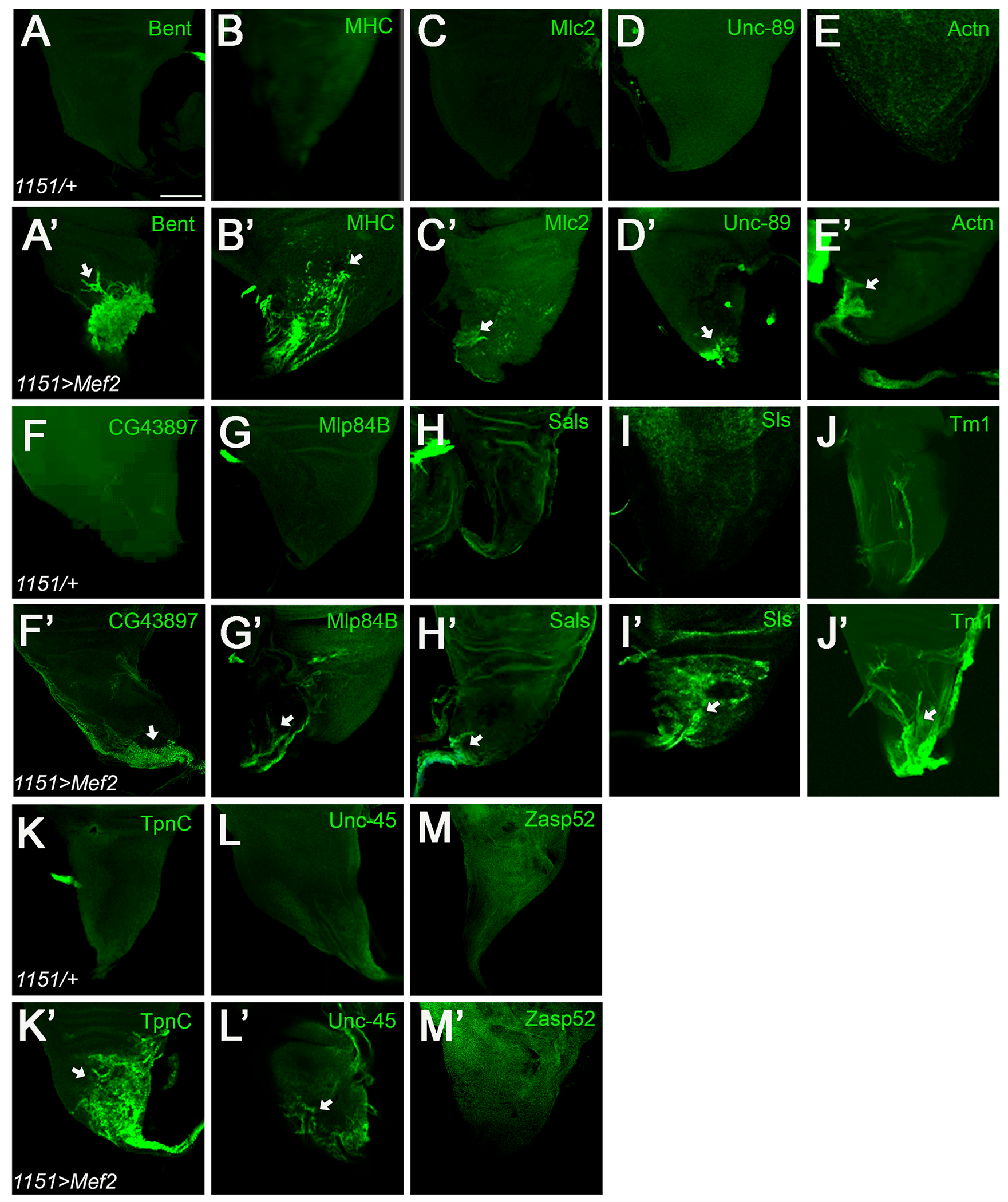
Immunofluorescence analysis of late third instar wing discs with elevated *Mef2* expression. All control *1151*/+ did not show accumulation of the tested polypeptides, whereas many polyppetides were observed to accumulate in control discs. (A-A′) Bent (Bt) is associated with the myosin thick filaments. (B-B′) Myosin heavy chain (MHC) accumulates in myosin thick filaments and was used to visualize muscle differentiation. (C-C′) Myosin light chain 2 (Mlc2) accumulates in the myosin II complex of the myofibril. (D-D′) Obscurin (Unc-89) is a titin-like protein of the sarcomere. (E-E′) α-actinin (Actn) is a Z-disc protein. (F-F′) CG43897 accumulates in the Z-disc. (G-G′) Mlp84B accumulates in the Z-disk. (H-H′) Sarcomere length short (sals) is a sarcomeric protein expressed on the striated muscle thin filament. (I-I′) Sallimus (Sls) links myosin filaments to the Z-disk and accumulates at the Z-disc. (J-J′) Tropomyosin (Tm1) binds along the actin thin filament. (K-K′) Troponin C (TpnC) is part of the troponin complex and is found along the actin thin filament. (L-L′) Uncoordinated 45 (unc-45) binds to myosin and accumulates along the thick filaments. (M-M′) Z band alternatively spliced PDZ-motif protein 52 (Zasp52) did not show accumulation on control and experimental wing discs. Scale bar: 50 μm.

To validate the up-regulation of myofibrillar protein genes, we obtained antibodies recognizing the products of 16 of the up-regulated genes. We chose to detect the products of genes that were both significantly up-regulated and those that were not, to assess how representative was the RNA-seq analysis. Three antibodies failed specificity testing (see Materials and Methods), meaning that we tested 13 different myofibrillar proteins for accumulation in control and experimental discs (Table 2 and Fig. 4). Fig. 4 shows representative control and experimental discs stained with these 13 antibodies, as discussed in more detail below. Table 2 summarizes the immunofluorescent analysis of the 13 polypeptides, where we quantify the percentage of discs that show protein accumulation in control and experimental samples, and the location of the protein product within normal myofibrils. In addition, we quantified the level of accumulation of each protein by calculating the fraction of the area covered by the stain, in relation to the myoblast pool. Fig. S14 shows the myoblast areas (as visualized using anti-MEF2) for each of the experimental discs shown here that demonstrates a roughly equivalent myoblast pool in all experimental discs. This observation emphasizes that differences in the extent of accumulation of different myofibrillar proteins, as shown below, does not arise from differences in the myoblast pool size.

Overall, we detected 12 of the 13 myofibrillar proteins in experimental wing imaginal discs but absent in control discs (Fig. 4). We found that the sarcomeric protein Bent (Bt) (Tanentzapf and Perkins, 2014), the motor protein MHC (Wells et al., 1996; Standiford et al., 1997), the contractile protein myosin light chain 2 (MLC2) (Warmke et al., 1992) and the titin-like protein Obscurin (unc-89) (Katzemich et al., 2015) showed protein products accumulating in the experimental discs (Fig. 4A–D’). For these four proteins, their transcripts were significantly up-regulated in the RNA-seq data compared to the control (Fig. 3D). The fraction of myoblast area covered by each of these stains ranged from 0.8% for Unc-89 to 54% for MHC (Table 2).

For eight proteins (α-actinin (Actn), CG43897, Muscle LIM protein at 84B (Mlp84B), Sarcomere length short (Sals), Sallimus (Sls), Tropomyosin (Tm1), Troponin C (TpnC) and Unc-45, we also observed significant accumulation in experimental but not control discs (Fig. 4E–L’), even though their transcripts were not significantly up-regualted in the RNA-seq dataset. Actn aids in actin filament binding (Gaudet et al., 2011), CG43897 is predicted to be involved in mesoderm development (Furlong et al., 2001), Mlp84B maintains muscle structural integrity (Clark et al., 2007), Sals promotes the elongation of actin filaments during muscle growth (Bai et al., 2007), Sls helps attach myosin filaments to the Z-disc (Burkart et al., 2007), Tm1 regulates muscle contraction (Gaudet et al., 2011), TpnC is required for myofibril assembly (Chechenova et al., 2017), and Unc-45 aides in myosin folding (Lee et al., 2011). The fraction of myoblast area covered by each stain ranged from 28.18% for TpnC to 1.66% for Unc-45 (Table 2), indicating that some genes were more responsive to activation by MEF2 than others.

Finally, Z band alternatively spliced PDZ-motif protein 52 (Zasp52), which accumulates in the Z-disc of developing myofibrils and attaches Actn to the Z-disc (Jani and Schock, 2007; Gaudet et al., 2011), was not significantly up-regulated in the RNA-seq data (Fig. 3D) and did not show protein accumulation in control nor experimental discs (Fig. 4M–M’). To test whether the lack of staining was attributed to a faulty Zasp52 antibody, we conducted cryosectioning and immunostaining of *w^1118^* adult thoracic muscles. This analysis revealed staining for Zasp52, suggesting that this antibody is working (data not shown), however, this antibody likely recognizes an adult-specific isoform (Chechenova et al., 2013).

Taken together, these immunofluorescent studies broadly support the RNA-seq data in demonstrating that the products of up-regulated myofibrillar protein genes are detected in *Mef2*-over-expressing myoblasts. Nevertheless, the activation of the myogenic program is incomplete, at least at this stage of development.

### 3.4. Up-regulated genes are characteristic of embryonic and larval muscle rather than pupal and adult muscle

The adult skeletal muscles of *Drosophila* are more complex than their larval counterparts (Miller, 1950), and this complexity is reflected in part by the existence of several muscle protein genes that are expressed in the pupal and adult muscles but not in the embryonic and larval stages, and several genes that are differentially spliced between larval and adult muscles (reviewed in Bernstein et al., 1993).

Since the imaginal myoblasts are precursors of the adult muscles, we investigated whether *Mef2* activates a larval or adult myogenic program, by mining the RNA-seq data for genes known to be expressed either at all stages of muscle development or just in the developing adult muscles.

**Fig. 5.**
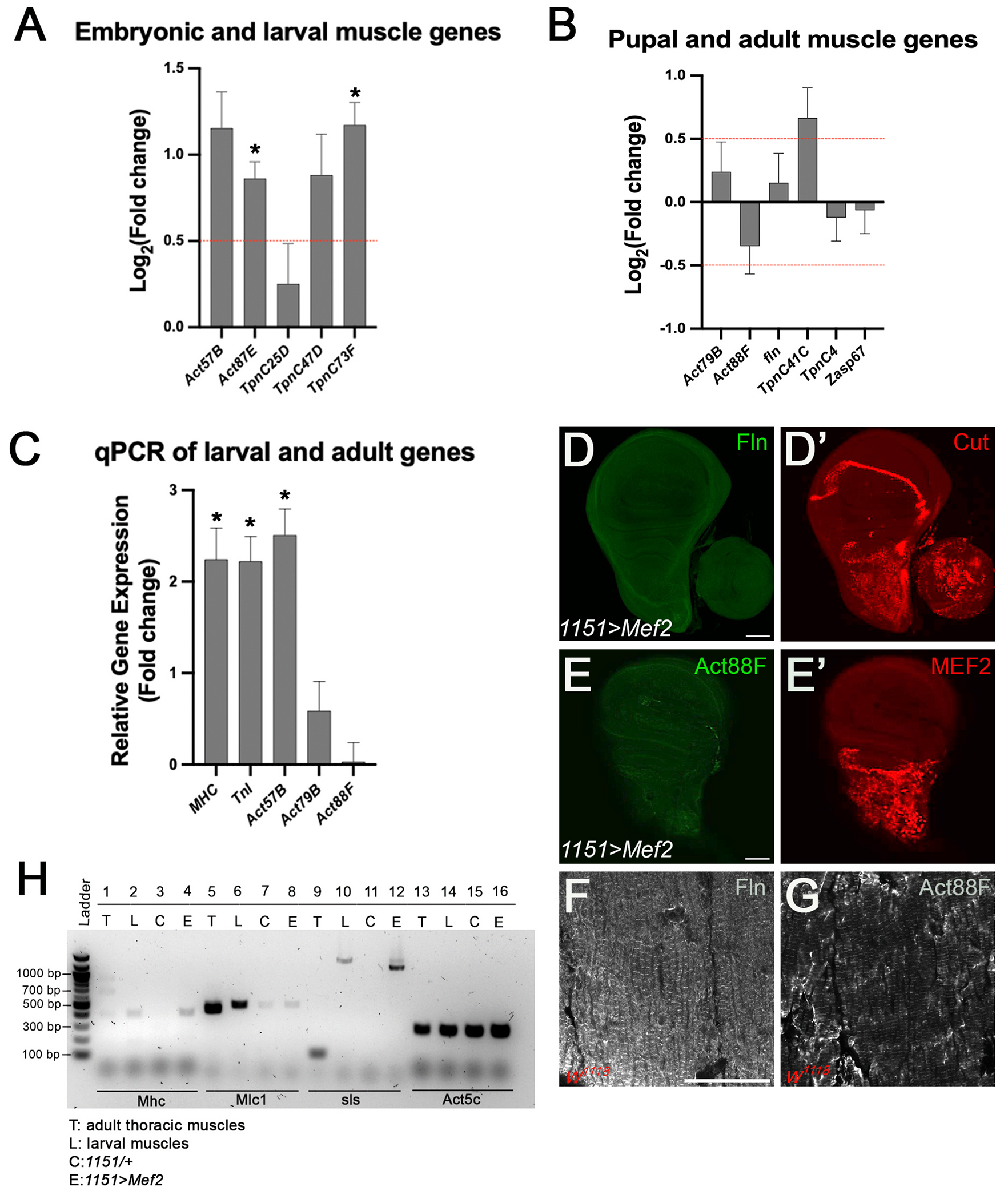
Up-regulated genes are characteristic of embryonic and larval muscle. (A–B) Bar graphs showing genes associated with embryonic and larval muscle, and pupal and adult muscles. Significant differentially expressed genes (DEGs) in wing imaginal discs of late third-instar female larvae upon *Mef2* over-expression are indicated with an asterisk. Log_2_ fold change greater than or less than 0.5 plotted against p-value <0.05 was deemed significant (red dotted line). The embryonic and larval muscle genes, *Act87E* and *TpnC73F*, were significantly up-regulated. No significant DEGs were observed for the pupal and adult muscle genes category. (C) Quantitative RT-PCR (qPCR) of larval and adult genes revealed *Mhc*, *TnI*, and *Act57B* to be significantly up-regulated. Log fold change greater than two was deemed significant (asterisk). (D–E) Immunofluorescence of late third instar female wing discs with elevated *Mef2* expression. *flightin* (*fln*) and *Actin88F* (*Act88F*) did not show expression in experimental wing discs. (D′-E′) Cut and MEF2 stains show the myoblast pools for each disc. Scale bar: 50 μm. (F–G) Immunofluorescence of 1–2 day old female thoracic indirect flight muscles (IFMs). Fln and Act88F showed accumulation in the IFMs. Scale bar: 50 μm. (H) RT-PCR of *Mhc*, *Mlc1*, and *sls in w*^*1118*^ female adult thoracic muscles (T), *w*^*1118*^ whole larval muscles (L), control wing discs (C), and experimental wing discs (E) revealed larval isoforms to be expressed in experimental wing discs. *Act5C* was used as a loading control. Lane numbers are depicted on the first row. *Mhc* exon 18 is included in T (907 bp; lane 1) but excluded in L and E (411 bp; lanes 2 and 4), and C revealed no bands (lane 3). *Mlc1* exon 5 is excluded in T (447 bp; lane 5) but included in L, C, and E (494 bp; lanes 6, 7, and 8). For *sls,* exon 10 is excluded in T (106 bp; lane 9) and included in L and E (1075 bp; lanes 10 and 12), and C revealed no bands (lane 11). All graphs are Mean ± Standard Error (Student’s two-tailed *t*-test).

For muscle actin, *Act57B* and *Act87E* are expressed throughout the life cycle, whereas *Act79B* and *Act88F* expression are specific to the adult muscles (Fyrberg et al., 1983; Sanchez et al., 1983). In the RNA-seq data, expression of both *Act57B* and *Act87E* were up-regulated, the latter significantly (Fig. 5A); by contrast *Act79B* was only minimally up-regulated (and not significantly), and *Act88F* expression was down-regulated (not significantly) (Fig. 5B).

For muscle Troponin C, *TpnC25D, TpnC47D,* and *TpnC73F* are expressed throughout the life cycle, whereas *TpnC4* and *TpnC41C* expression are specific to the adult muscles (Herranz et al., 2004; Chechenova et al., 2017). In the RNA-seq data, expression of *TpnC25D, TpnC47D,* and *TpnC73F* were all up-regulated, the latter significantly (Fig. 5A); for the adult TpnC genes, *TpnC41C* expression was up-regulated (not significant) and *TpnC4* expression was slightly down-regulated (not significant) (Fig. 5B).

There are also a small number of known adult-specific myofibrillar protein genes, including *flightin* (*fln* (Vigoreaux et al., 1993) and *Zasp67* (Katzemich et al., 2013). Neither of these genes was significantly up-regulated in the experimental dataset (Fig. 5B).

To provide validation for some of these findings, we carried out quantitative RT-PCR (qPCR) of experimental and control RNA samples, performed in triplicate for each genotype. First, we confirmed the robust and significant up-regulation of *Mhc* and *TnI* in experimental RNA samples compared to controls (Fig. 5C). Next, we assessed the expression of *Act57B*, *Act79B*, and *Act88F*. Only *Act57B,* which is expressed throughout the life cycle, showed significant upregulation in response to MEF2.

We also stained wing imaginal discs for the accumulation of Fln (*1151*>*w^1118^* n = 10; *1151*>*Mef2* n = 8) and Actin88F (*1151*>*w^1118^* n = 11; *1151*>*Mef2* n = 11) (Fig. 5D–E’), and we validated Fln and Actin88F antibodies by immunostaining the thorax of 1-day old *w^1118^* female flies (Fig. 5F–G). In support of the RNA-seq and qPCR assays, antibodies recognizing specifically Fln and Act88F did not stain experimental wing imaginal discs, although sections of adult muscles stained robustly with each antibody (Fig. 5F–G, Table 2).

To further analyze whether *Mef2* activates a larval or adult myogenic program, we assessed the patterns of alternative RNA splicing for genes that are differentially spliced between the flight muscles and larval muscles (Bernstein et al., 1993; Oas et al., 2014). In the RNA-seq dataset, only one gene encoding a splicing factor was differentially expressed. This gene, *smooth,* which was down-regulated, but it is not known to be expressed in the mesoderm. We used primers for RT-PCR that flanked specific regions within *Mhc*, *Mlc1*, and *sls* that show differential splicing between muscle types (Oas et al., 2014). We performed RT-PCR using RNA extracted from *w^1118^* female adult thoracic muscles (T), *w^1118^* whole larval muscles (L), control wing discs (C), and experimental wing discs (E) (Fig. 5H). *Act5C* was used as a loading control. In the adult thoracic flight muscles, MHC exon 18 is included but excluded in other muscles. So, we designed a forward primer in exon 17, and a reverse primer in exon 19 to analyze this difference between control and experimental samples (Bernstein et al., 1986; Hastings and Emerson, 1991). As expected, we saw a band of 907 bp for the thoracic muscle sample (Fig. 5H; lane 1) and no bands for the control experimental disc sample (Fig. 5H; lane 3). We also observed that the experimental disc sample excludes exon 18 (Fig. 5H; lane 4), similar to the larval muscle sample (Fig. 5H; lane 2).

Next, we assessed *Mlc1* transcripts, which differ between larval and adult transcripts by 47 nucleotides, because *Mlc1* exon 5 is excluded in thoracic flight muscles but included in larval muscles (Falkenthal et al., 1987). For the thoracic muscle sample, we saw a band at 447 bp (Fig. 5H; lane 5) and a band at 494 bp for the larval muscle sample (Fig. 5H; lane 6). For imaginal discs, we observed bands at 494 bp in both control and experimental disc samples (Fig. 5H; lanes 7 and 8), similar to what was observed for the larval muscle sample.

Lastly, we analyzed *sls*, which encodes a Titin isoform. Transcripts of the *sls* gene exclude exon 10 in flight muscles, whereas it includes this exon in jump muscles (Oas et al., 2014). We observed a band size of 106 bp for the thoracic muscle sample (Fig. 5H; lane 9) and a band size of 1075 bp for the larval muscle sample ((Fig. 5H; lane 10). The control wing disc sample did not reveal a band (Fig. 5H; lane 11) but the experimental wing disc sample revealed two bands, one of which was at 1075 bp, similar to the larval muscle sample, and the other band was slightly smaller (Fig. 5H; lane 12).

Overall, we conclude that the predominant myogenic splicing program that is up-regulated in wing imaginal discs more closely reflected the embryonic/larval program, rather than the formation of the more complex pupal/adult muscles. Interestingly, whereas two out of three myofibrillar protein genes assayed did not show transcripts in the control discs, we observed low levels of *Mlc1* transcripts in control discs. This suggests that some aspects of the myogenic program may be poised to be activated even in control discs, and is consistent with the detection of *Act57B* transcripts in wild-type imaginal discs (Butler et al., 2003).

## 4. Discussion

In this paper, we found that enhanced expression of *Drosophila Mef2* in adult myoblasts of the larval wing imaginal discs induced an incomplete myogenic response. Transcriptomic analysis, coupled with immunofluorescence and qPCR, of whole wing discs from control and experimental female larvae revealed a preference for the expression of muscle structural genes. Genes most strongly activated tended to be those expressed throughout normal development, rather than those genes specific for the pupal or adult stages.

### 4.1. Heterogeneity in muscle differentiation stages

Immunofluorescent analysis of discs displaying muscle differentiation revealed striations in certain myofibril-like structures, whereas others lacked this characteristic. One interpretation of this observation is that the two different phenotypes represent successive stages of myofibril assembly occurring, beginning with the accumulation of myofibrillar proteins, and subsequent assembly into ordered structures. Myofibril assembly is thought to occur first through the accumulation of alpha-actinin and titin into Z-bodies, and subsequent assembly into pre-myofibrils containing mostly myofibrillar components plus non-muscle myosin (encoded by zipper in *Drosophila*). Muscle myosin subsequently displaces the non-muscle isoform as the myofibrils mature (Sanger et al., 2002; reviewed in Sparrow and Schöck, 2009). In this scenario, the un-ordered deposits of muscle proteins may be awaiting assembly. However, during normal pre-myofibril formation, these proteins are generally present at much lower levels. Furthermore, given the presence of muscle MHC both in deposits and in striated structures, it seems more likely that the deposits represent proteins that have failed to organize normally, perhaps because the normal sequential expression of myofibrillar protein genes is not occurring in this system.

Why, then, do some regions of the disc show organized structures whereas others do not? We imagine that the timing and level of accumulation of different myofibrillar proteins is somewhat stochastic between different cells, and it is possible that some proteins need to be present at significant levels to trigger ordered assembly. In this case, such “organizer” proteins should be more frequently present in striated structures and never in un-ordered deposits. Along these lines, we note that CG43897 and Zip almost exclusively accumulated in striated structures, suggesting either that they have organizer properties in the nascent myotubes, or are closely associated with proteins that may organize myofibril assembly.

### 4.2. MEF2 accumulation and differentiation varied between myoblasts

Both MEF2 accumulation and the region of differentiation varied across the myoblast field: muscle differentiation more prominently occurred at the proximal region of the imaginal wing disc, where MEF2 accumulation was also more pronounced. The explanation for this regional localization is not clear, although Cut protein is known to repress differentiation and shows reduced expression in proximal myoblasts as a result of Wingless signaling repressing Cut accumulation (Sudarsan et al., 2001). Therefore, it is possible that differentiation occurs as a product of distance from higher *cut* expression, and differentiation might be potentiated by simultaneously knocking down *cut* expression. Variation in MEF2 accumulation might arise in myoblasts that are activated earlier for differentiation, and along these lines, MEF2 is known to activate its own expression in maturing muscles (Cripps et al., 2004), therefore those cells showing higher MEF2 accumulation might have activated this positive autoregulatory loop.

We also note the fundamental differences in detection that we are using when assessing transcript levels and protein accumulation. Are transcripts for a particular gene up-regulated in all myoblasts, or in just a subset of myoblasts? Since *Mef2* expression is controlled directly by the *1151-Gal4* driver, it must be up-regulated in all myoblasts, even though its expression appears to be further potentiated in a subset of myoblasts. For myofibrillar protein genes, it is as yet unclear if their expression is increased in all myoblasts or in just a subset of them. If the former, this would raise the interesting possibility that translational efficiency may differ between myoblasts. This question could partially be addressed by comparing protein accumulation with transcript levels using *in situ* hybridization.

### 4.3. Failure of MEF2 to induce myoblast fusion

Our results pose another question: why is myoblast fusion rarely observed, despite the knowledge that fusion genes are MEF2 targets? Our RNA-seq data analysis of fusion gene expression showed that only *m-spondin* (*mspo*) is significantly up-regulated, however, expression of other fusion genes that are known MEF2 targets such as *sns*, *rols*, *mbc*, and *kirre* were not significantly altered. It is possible that MEF2 requires the function of additional co-factors to trigger broad expression of myoblast fusion genes, and these co-factors are not activated by enhanced levels of MEF2. One likely candidate is the C2H2 zinc finger transcription factor Lameduck, which is both required for myoblast fusion and upstream of *Mef2* (Duan et al., 2001; Ruiz-Gomez et al., 2002; Cunha et al., 2010). It is also possible that other factors expressed in the myoblasts may function to suppress myogenesis (including myoblast fusion). These include the known myogenic repressor, Zfh1 (Postigo et al., 1999).

### 4.4. Confirmation of RNA-sequencing data

One source of variability in our data was the significance of gene expression changes when comparing RNA-seq and qPCR datasets: some genes whose expression was not significantly altered in the RNA-seq showed significant changes in gene expression by qPCR, and vice versa. In addition, we also observed several gene products accumulating in the imaginal discs despite transcripts of the parent gene not being significantly up-regulated. The first obvious explanation for this difference could be the low number of replicates. For both datasets, we used three biological replicates, which have a poorer statistical power than a larger set. Statistically speaking, obtaining more replicates will decrease random variability and improve reproducibility. A paper published by Schurch et al. (2016) used a full set of 42 biological replicates, in each of two conditions, to evaluate how many replicates are needed to identify significantly differentially expressed (SDE) genes. They found that when using DESeq2, at least 12 replicates should be used to identify SDE genes for 0.5- and 2.0-fold changes. In this study, we used DESeq2 and three replicates to identify SDE genes for 0.5-fold changes, therefore some level of false positives or false negatives could have arisen. On the other hand, RNA-seq was a valuable guide to identifying many genes whose changes in expression were validated either by qPCR or by immunofluorescence.

We also postulate that differences in normalization methods and data analysis pipelines used for RNA-seq and qPCR may contribute to this variability. The normalization process, using DESeq2 for RNA-seq analysis, adjusts variations by dividing the counts for each gene by the size factor for that sample. This method of normalization ensures that the counts are adjusted for differences in library size before performing a log transformation of the counts. On the other hand, qPCR relies on reference genes for normalization. The choice of reference genes is crucial and is typically validated during the experimental design, as was done in this experiment. Nevertheless, a consistent trend emerges from our data set, that being the ability of MEF2 to preferentially activate the larval myogenic program.

### 4.5. Activation of embryonic and larval versus pupal and adult genes

The adult muscles of *Drosophila* are more complex than those of the larva, and in particular, the thoracic muscles show at least two distinct fiber types that are not present in larval muscles (reviewed in Bernstein et al., 1993). Since the wing disc myoblasts are the precursors of these complex adult muscles (Bate et al., 1991; Fernandes et al., 1991; Sudarsan et al., 2001) we tested whether the muscle differentiation that occurred was reflective of adult muscles or larval muscles. Based upon the RNA-sequencing, qPCR, PCR, and immunofluorescence analysis, there was no strong evidence of up-regulation of adult muscle genes or splice variants. This suggests that MEF2 appears to be more active in driving the expression of genes required for muscle development during embryonic and larval stages rather than the pupal and adult stages. As the organism transitions to the pupal and adult stages, the role of MEF2 may evolve, requiring other regulatory factors to take the lead in driving the expression of genes essential for mature muscle tissue maintenance and function. Along these lines, of the 147 genes that were up-regulated, 27% had nearby MEF2 binding sites, as determined using embryonic ChIP-seq. By contrast, none of the adult muscle genes had adjacent MEF2 binding sites in the same data set, even though *TpnC41C* is a known MEF2 target (Chechenova et al., 2015). While these data are derived from MEF2 binding in embryos, they provide some guidance as to why MEF2 enrichment did not activate the adult myofibrillar protein genes that we analyzed.

Adult flight muscle identity is dependent upon a regulatory suite of factors including Spalt-major (Schönbauer et al., 2011), Extradenticle plus Homothorax (Bryantsev et al., 2012b), and H15 (Katti et al., 2022), most of which commence expression early during pupal development. Therefore, it is likely that the myoblasts of the wing disc have not yet assumed an adult-like identity, and myotubes arising from these myoblasts follow a default larval pattern of differentiation. It remains to be determined if the expression of these adult myogenesis factors can trigger adult patterns of gene expression in the naive wing disc myoblasts.

## Supplementary Material

Trujilo et al 2024 supp

## Figures and Tables

**Table 1:** Summary of RNA-sequencing statistics and mapping results. Each replicate was mapped to the *D. melanogaster* Dm6 reference genome. All sequences were single-end reads. The average read-length was 101 nt.

Library	% GC	Number of raw reads	Number of cleaned reads	Mapping rate
***1151/***+ **replicate 1**	47	30178968	28517245	95.26%
***1151/***+ **replicate 2**	48	31487326	29978815	95.65%
***1151/***+ **replicate 3**	48	32904070	30983815	95.50%
***1151*>*Mef2* replicate 1**	50	40241817	38715150	94.88%
***1151*>*Mef2* replicate 2**	48	32332760	30806108	95.24%
***1151*>*Mef2* replicate 3**	48	30027650	28541651	95.44%

**Table 2:** Polypeptide location in the myofibril and quantification of immunofluorescent stains of wing imaginal discs.

Polypeptide	Location in myofibril	Stain in 1151*>*w^1118^ (%)	Stain in 1151*>*Mef2 (%)	^a^Fraction of myoblast area covered by stain (% ± SEM)
**Significantly up-regulated and stained**				
**Bent**	Z-disc	0/8 (0%)	9/9 (100.00%)	37.24% ± 6.68
**MHC**	A band	0/20 (0%)	11/11 (100.00%)	54.74% ± 8.54
**MLC2**	Myosin filament	0/8 (0%)	6/8 (75.00%)	12.63% ± 4.64
**Unc-89/** **Obscurin**	M-line	0/10 (0%)	4/13 (30.77%)	0.80% ± 0.94
**Significantly up-regulated, but did not stain (likely antibody not working)**				
**Mf**	A band	0/15 (0%)	0/8 (0%)	0%
**MLC1**	Myosin filament	0/13 (0%)	0/12 (0%)	0%
**TnT/up**	Actin filament	0/12 (0%)	0/9 (0%)	0%
**Not significantly up-regulated, but stained**				
**Actn**	Z-disc	0/8 (0%)	7/7 (100%)	17.44% ± 5.68
**CG43897**	Z-disc	0/7 (0%)	5/8 (62.5%)	7.26% ± 2.92
**Mlp84B**	Z-disc	0/10 (0%)	8/15 (53.33%)	2.46% ± 0.82
**Sals**	Actin filament	0/10 (0%)	7/9 (77.78%)	8.88% ± 3.52
**Sls**	Z-disc	0/8 (0%)	7/9 (77.78%)	6.88% ± 2.47
**Tm1**	Actin filament	0/9 (0%)	8/10 (80%)	19.87% ± 11.62
**TpnC**	Actin filament	0/11 (0%)	12/14	28.18% ± 7.75 (85.71%)
**Unc-45**	Myosin filament	0/6 (0%)	3/9 (33.33%)	1.66% ± 1.49
**Not significantly up-regulated and did not stain**				
**Zasp52**	Z-disc	0/12 (0%)	0/13 (0%)	0%
**Adult-specific genes not significantly upregulated**				
**Fln**	Myosin filament	0/10 (0%)	0/8 (0%)	0%
**Act88F**	Actin filament	0/11 (0%)	0/11 (0%)	0%

a Proportion of the area containing protein accumulation compared to the total area of the myoblasts as revealed by MEF2 staining.

## Data Availability

Data will be made available on request.

## Appendix A. Supplementary data

Supplementary data to this article can be found online at https://doi.org/10.1016/j.ydbio.2024.08.004.
