# Supplementary material for: Enhanced expression of the myogenic factor Myocyte enhancer factor-2 in imaginal disc myoblasts activates a partial, but incomplete, muscle development program
Source: Dev Biol. Author manuscript; Available in PMC 2026 Feb 12. (PMC12898291; doi:10.1016/j.ydbio.2024.08.004)
Supplement: Trujilo et al 2024 supp [file NIHMS2137281-supplement-Trujilo_et_al_2024_supp.pdf]

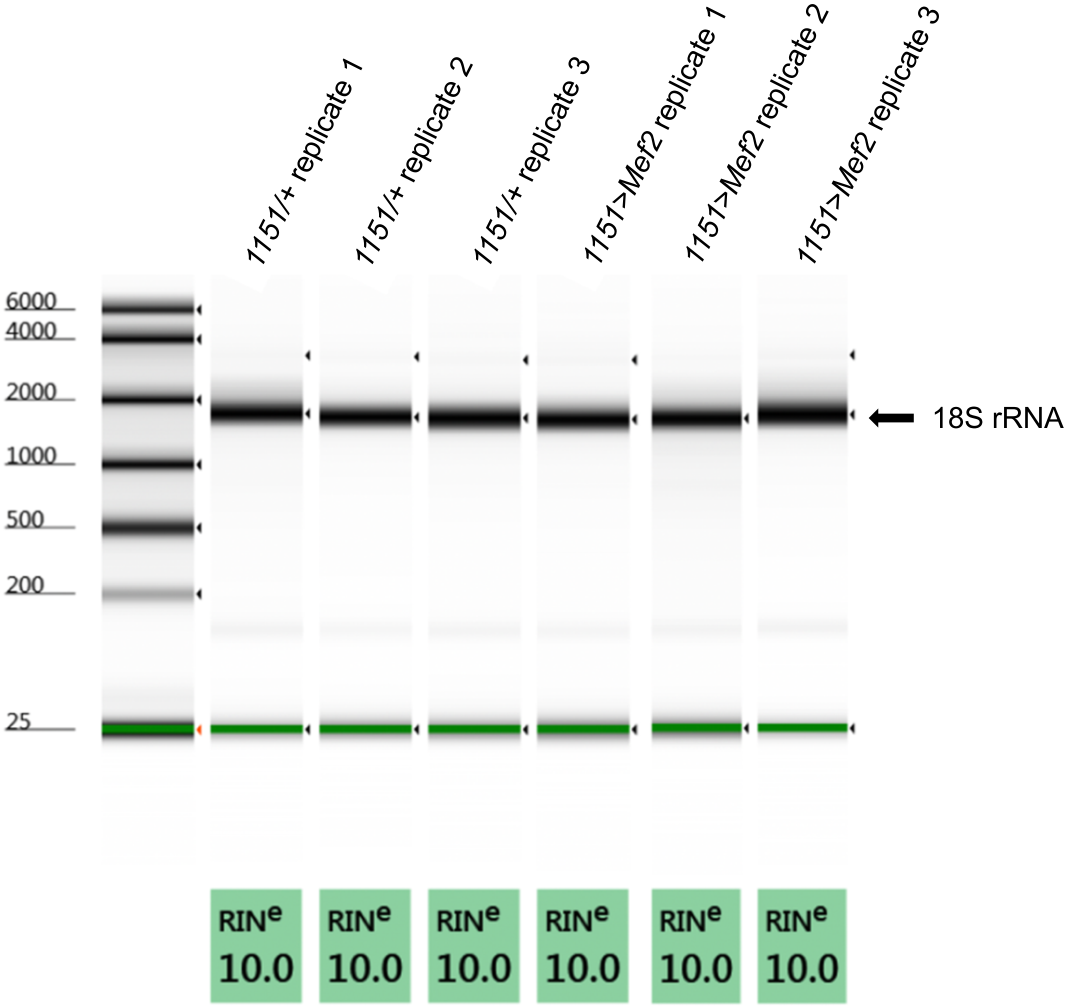

Figure S1. **RNA integrity number (RIN).** Electrophoresis gel of *1151/+* control replicates 1-3 and *1151>Mef2* experimental replicates 4-6 RNA integrity number (RIN). A RIN score of 10.0 indicates good quality RNA while 1.0 indicates poor quality RNA. In *Drosophila melanogaster*, the total RNA ratio is determined by dividing the area under the 18S rRNA peak by the total area under the graph. No RNA degradation, shown by RNA smearing below the 18S RNA band, is seen here, indicating good quality RNA.

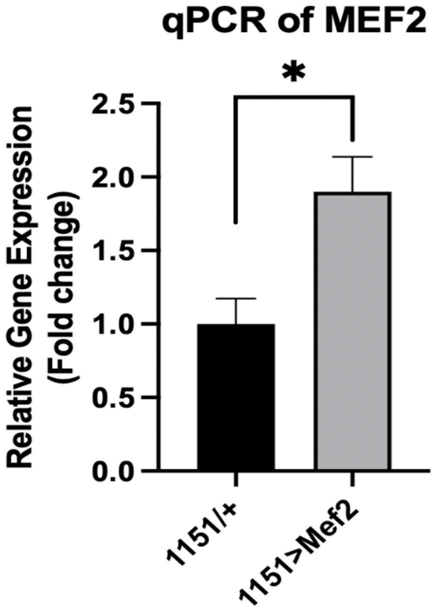

Figure S2. Quantitative RT-PCR (qPCR) of 1151/+ and 1151>Mef2 imaginal wing discs revealed *Mef2* to be up-regulated. The p-value is 0.02, which is significant. Significance is marked at a p-value below 0.05. The mean ± standard error of the mean was used for this graph.

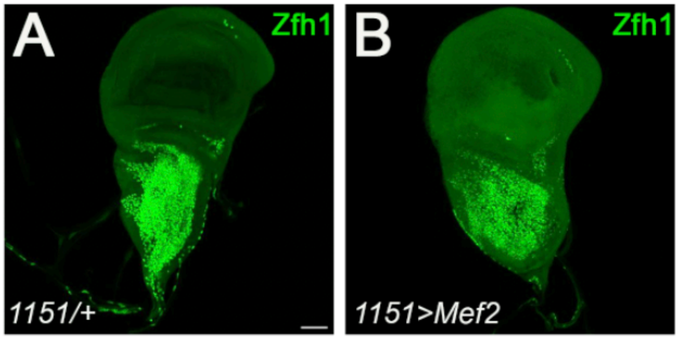

Figure S3. Phenotypic analysis of MEF2-induced myogenesis in wing discs reveals a reduction of Zfh1-positive cells. (A) Control *1151/+* and (B) experimental *1151>Mef2* discs of third instar larva upon *Mef2* over-expression stained against Zfh1 showed a reduction in the myoblast area occupied by Zfh1 positive cells. Control n=16. Experimental n=6. Scale bar: 50 μm.

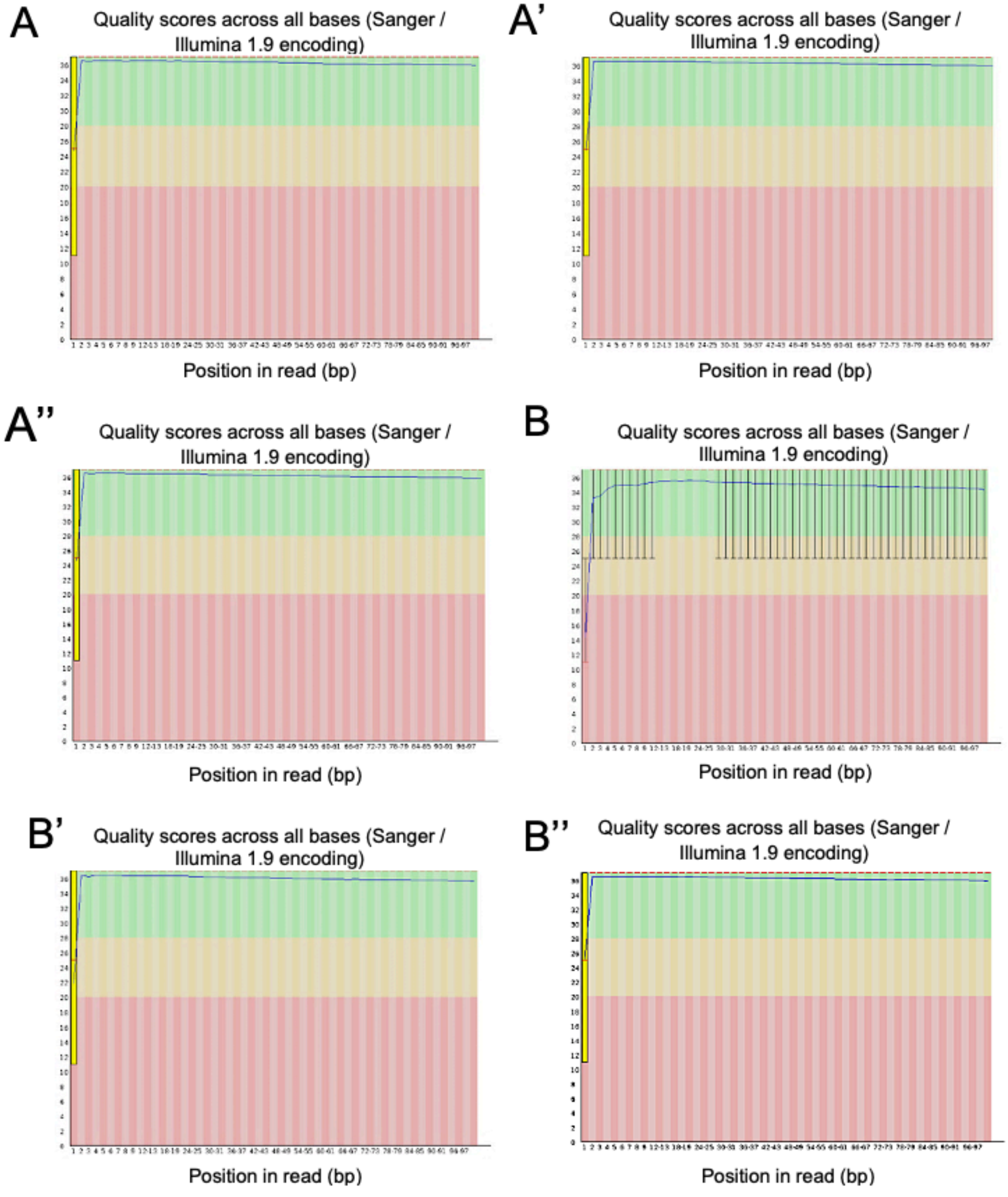

Figure S4. Per base sequence quality. The BoxWhisker plot shows the quality score distribution across all bases. The red line represents the median value and the blue line represents the mean quality. The yellow box represents the interquartile range from 25% to 75%. The upper whisker represents 10% points while the lower whisker represents 90% points. Here, base calls falling in the green area indicate a very good-quality call. (A-A’’) *1151/+* control replicates 1-3 (B-B’’) *1151>Mef2* experimental replicates 4-6.

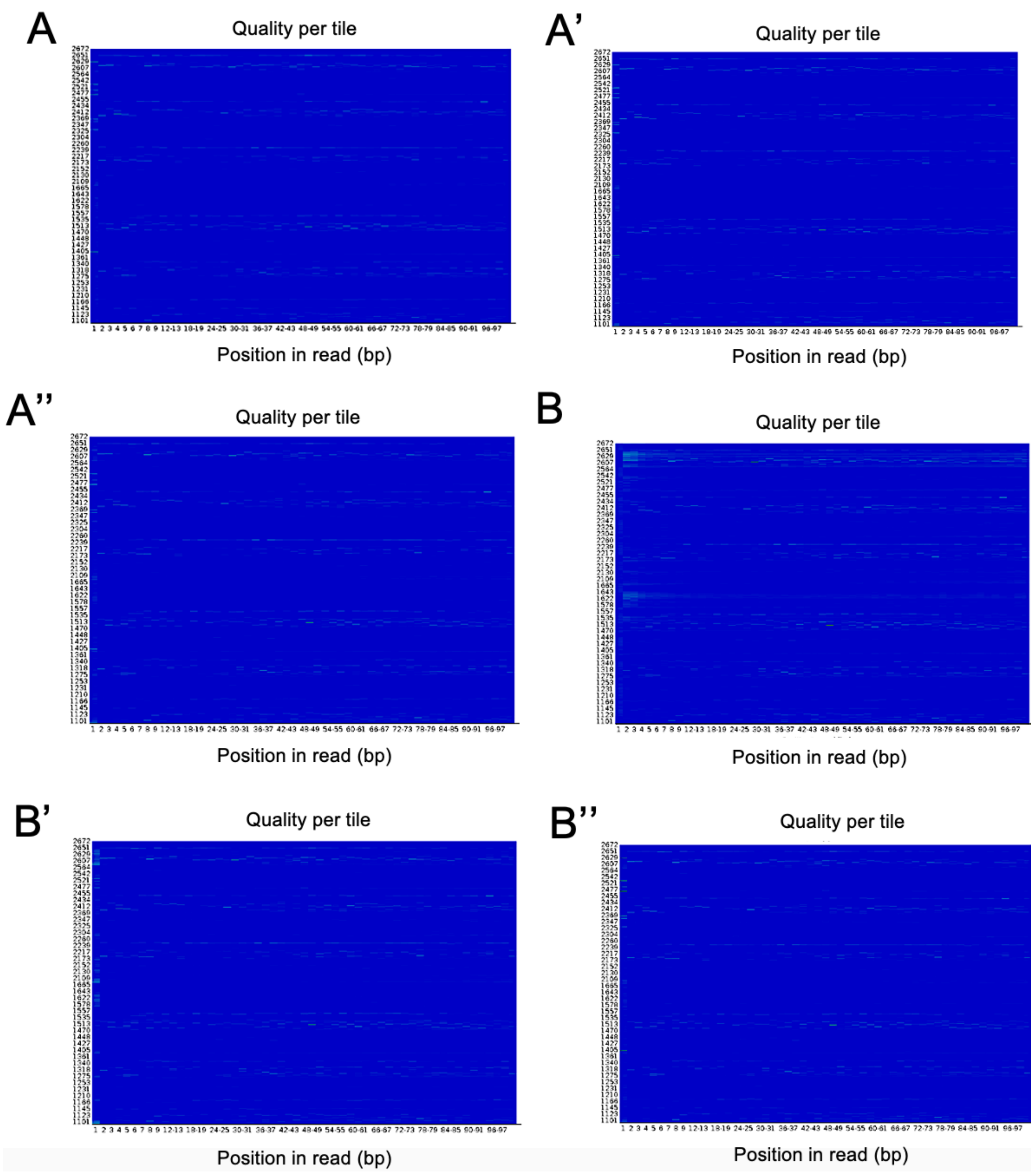

Figure S5. Per tile sequence quality. Per tile sequence quality shows the deviation from the average quality for each tile. Cool colors (blues) indicate where the quality was at or above the average for that base in the run. Hot colors (red) indicated where the quality was below the average for that base in the run. Like here, a good plot is blue all over. (A-A'') *1151/+* control replicates 1-3 (B-B'') *1151>Mef2* experimental replicates 4-6.

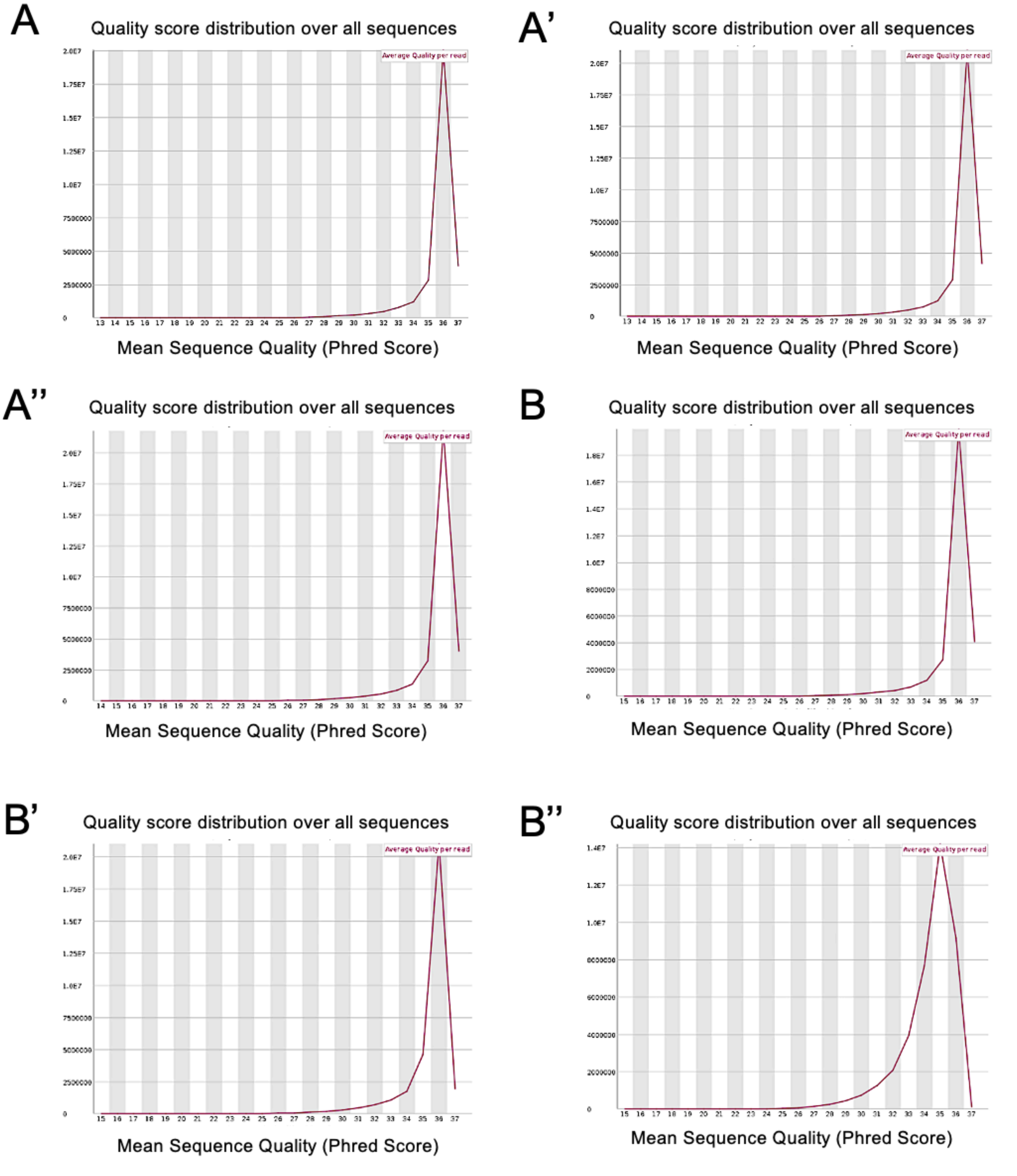

Figure S6. Per sequence quality scores. The most frequently observed mean quality is 35+ for all samples, representing good quality. (A-A’’) *1151/+* control replicates 1-3 (B-B’’) *1151>Mef2* experimental replicates 4-6.

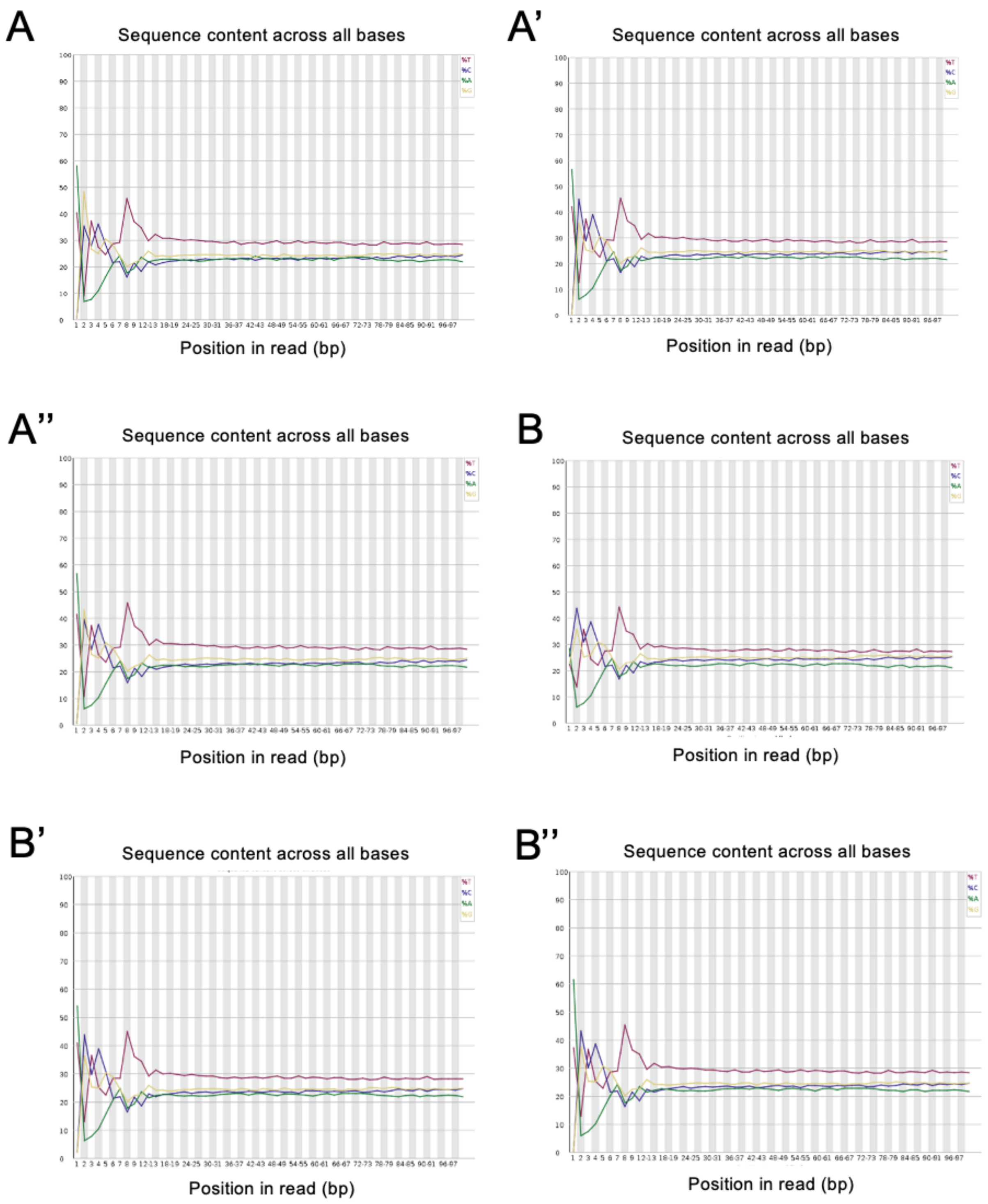

Figure S7. Per base sequence content. The different bases of the sequence run are not greatly imbalanced, indicating low bias. Libraries, such as these, have a selection bias in the first 12 base pairs of each run, but it does not represent individual biased sequences. Thus, the ability to measure expression is not affected. (A-A'') *1151/+* control replicates 1-3 (B-B'') *1151>Mef2* experimental replicates 4-6.

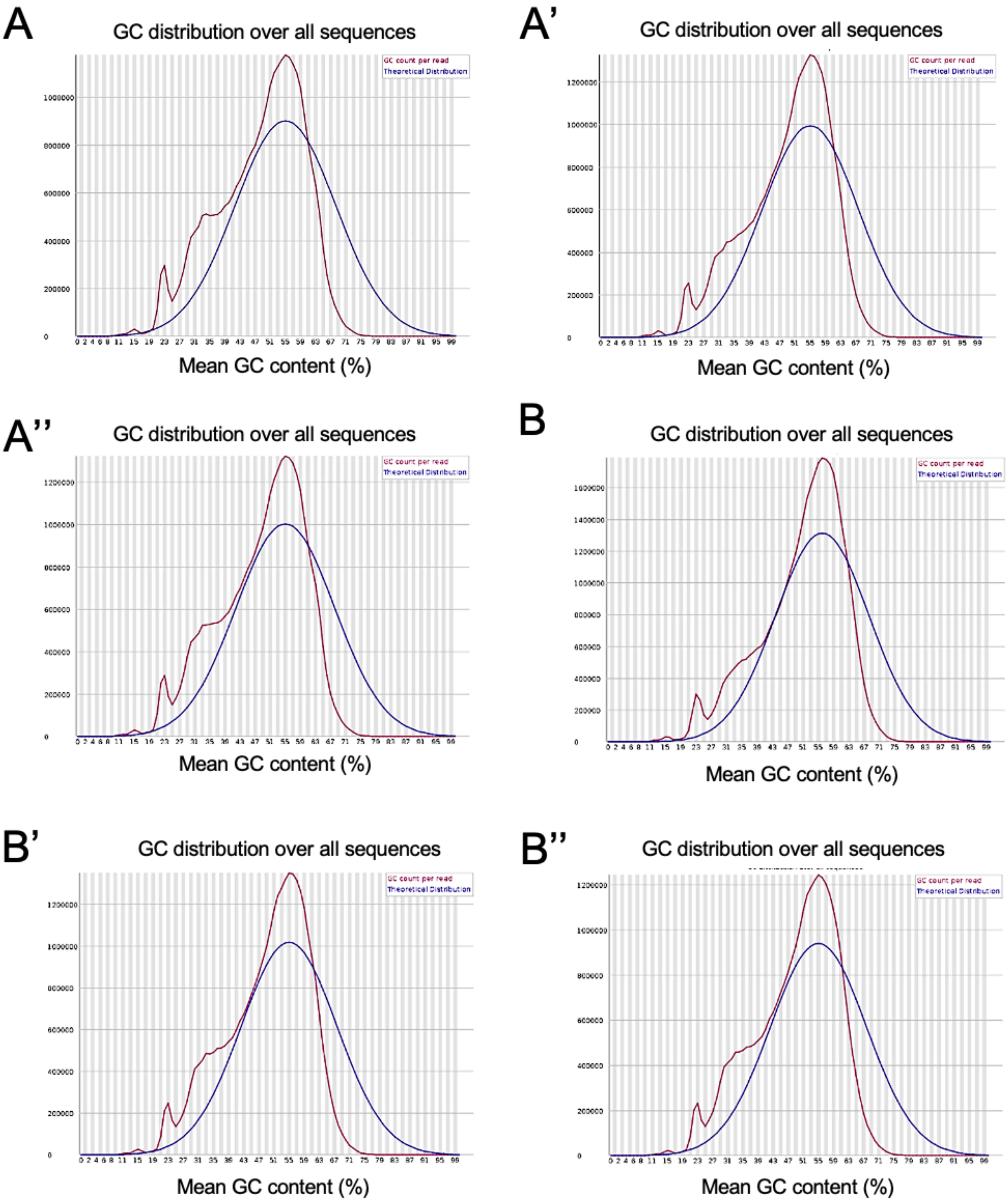

Figure S8. Per sequence GC content. The blue line represents the theoretical normal distribution of the GC content. The red line represents the distribution of the GC count per read. The distribution across all samples is near normal and falls within a good GC content range of 47%-50% for *Drosophila melanogaster*. (A-A'') *1151/+* control replicates 1-3 (B-B'') *1151>Mef2* experimental replicates 4-6.

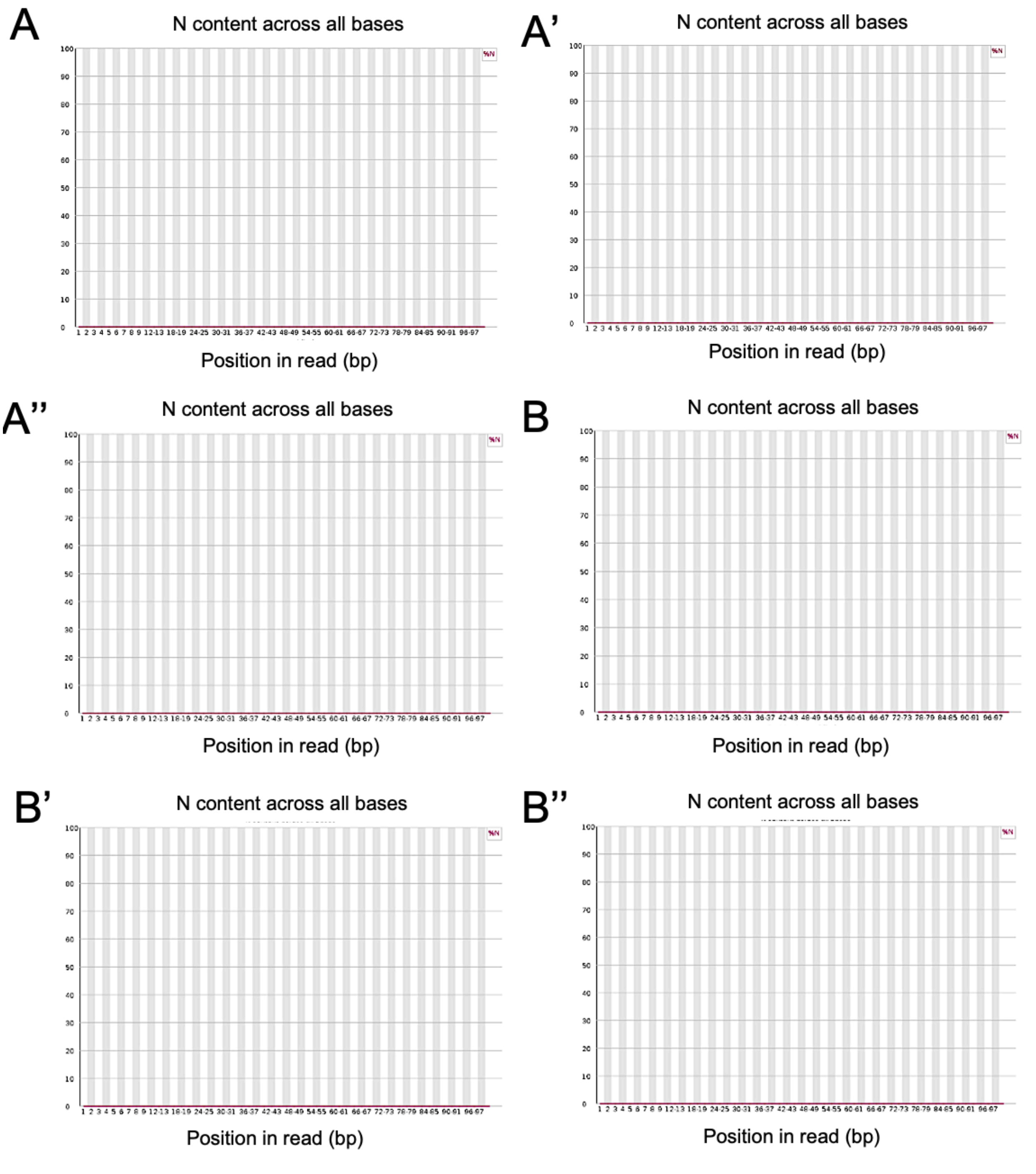

Figure S9. Per base N content. Per base N content shows the percentage of base calls at each position where N is called. All samples show a low proportion of Ns appearing in the sequence. This indicates that the Illumina sequencer was able to interpret the data well enough to make a base call with sufficient confidence. (A-A’’) *1151/+* control replicates 1-3 (B-B’’) *1151>Mef2* experimental replicates 4-6.

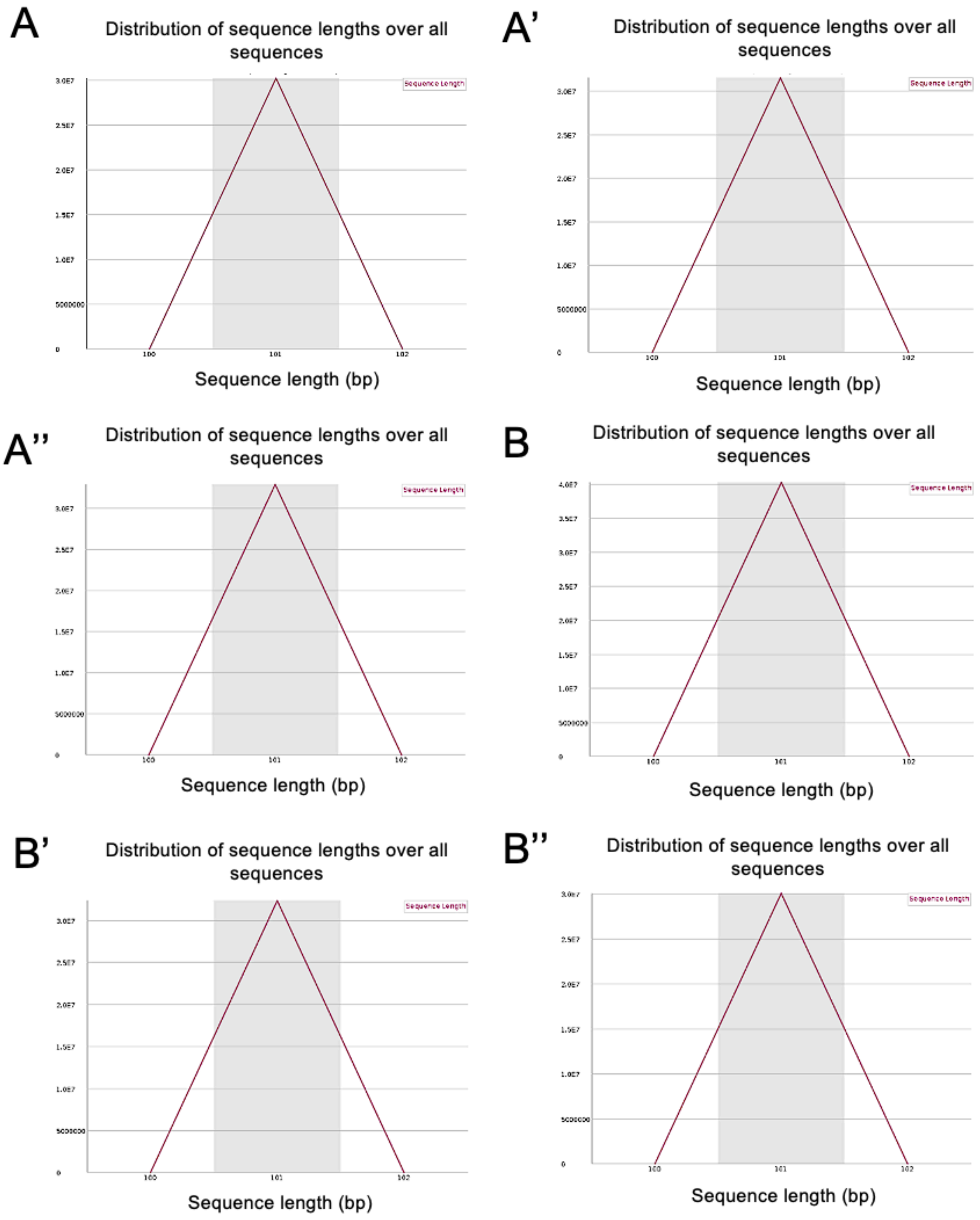

Figure S10. Sequence length distribution. The peak in these graphs shows a uniform length of 101 base pairs across all samples. This uniformity indicates that the Illumina sequencer produced good-quality sequence fragment lengths. (A-A'') *1151/+* control replicates 1-3 (B-B'') *1151>Mef2* experimental replicates 4-6.

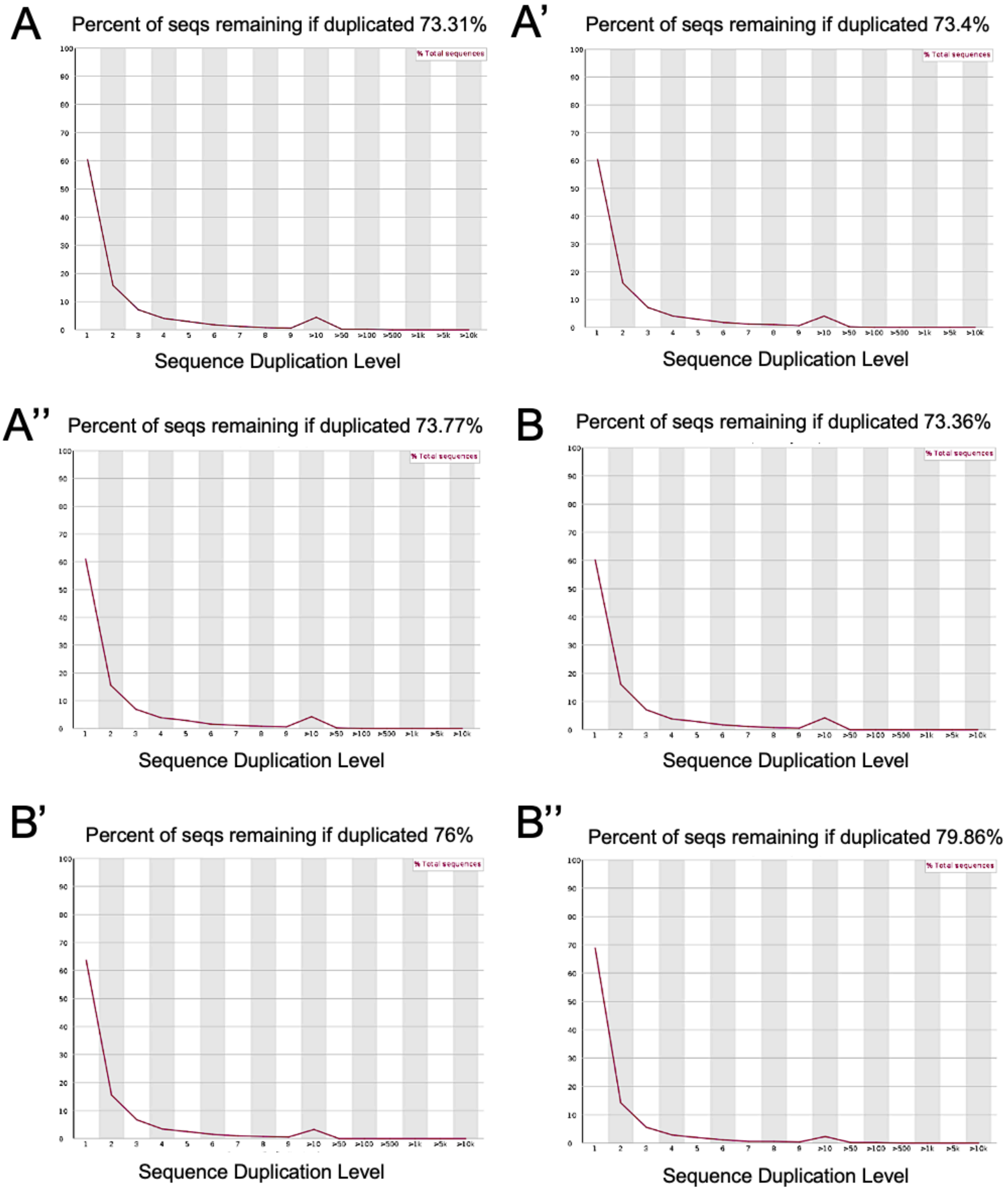

Figure S11. Sequence duplication levels. The red line represents the percent of total sequences. A duplication level near level 1 indicates that the library is diverse resulting in unique reads. Low duplication levels between 2-9 copies indicate reads derived from mRNA. Moderate duplication levels between 10-100 copies indicate reads are derived from rRNA and/or genomic regions that are highly repetitive. All samples show low duplication levels. (A-A'') *1151/+* control replicates 1-3 (B-B'') *1151>Mef2* experimental replicates 4-6.

Table S1. Overrepresented sequences. This library contains a diverse set of sequences with no individual sequence being overrepresented.

Library	Overrepresented Sequences
***1151/+* replicate 1**	None
***1151/+* replicate 2**	None
***1151/+* replicate 3**	None
***1151>Mef*2 replicate 1**	None
***1151>Mef*2 replicate 2**	None
***1151>Mef*2 replicate 3**	None

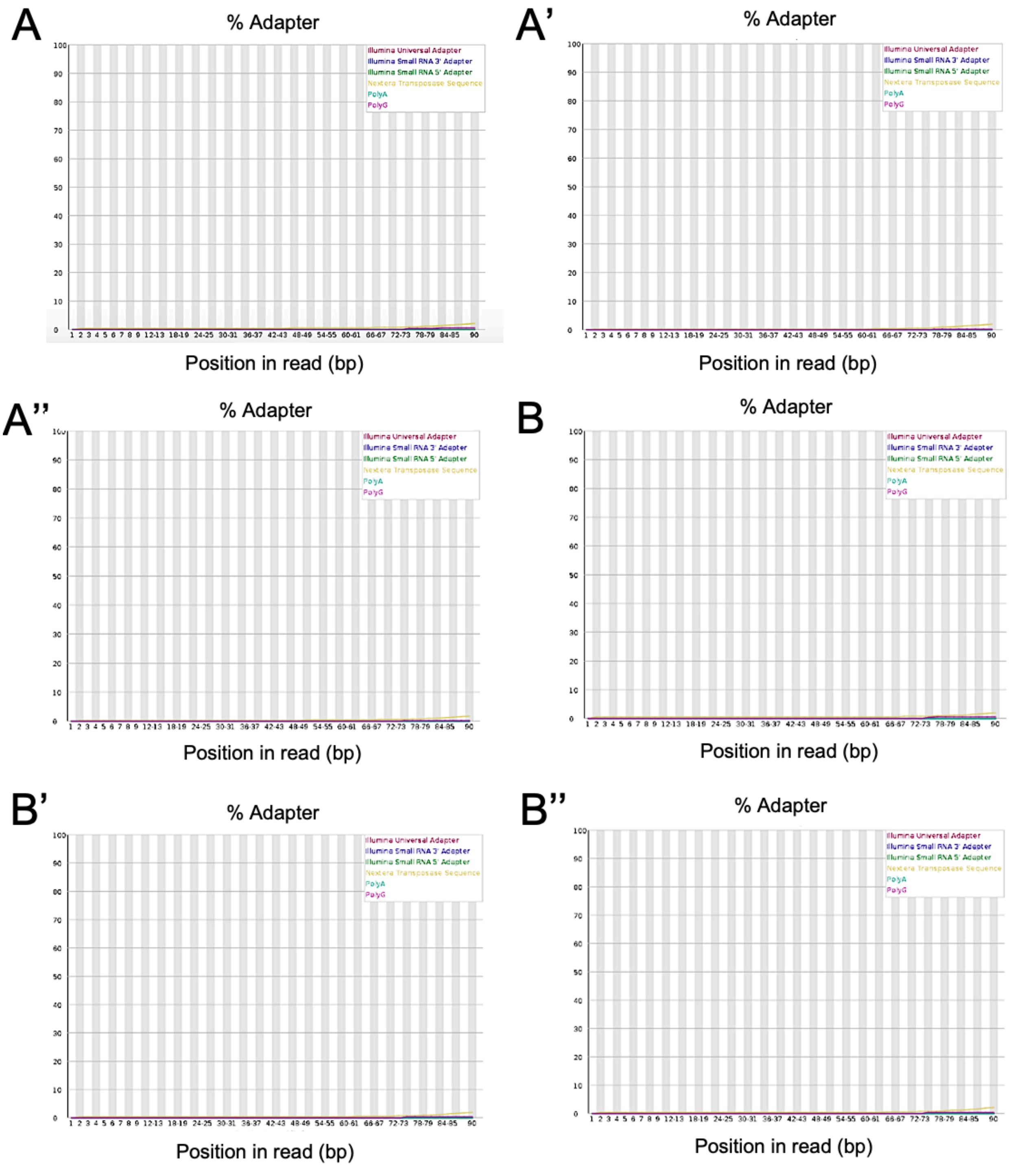

Figure S12. Adapter content. These graphs show the percentage of sequences that contain adapter sequences. The low percentage of adapter sequences in these graphs indicates no adapter contamination in the sequencing data. (A-A'') *1151/+* control replicates 1-3 (B-B'') *1151>Mef2*.

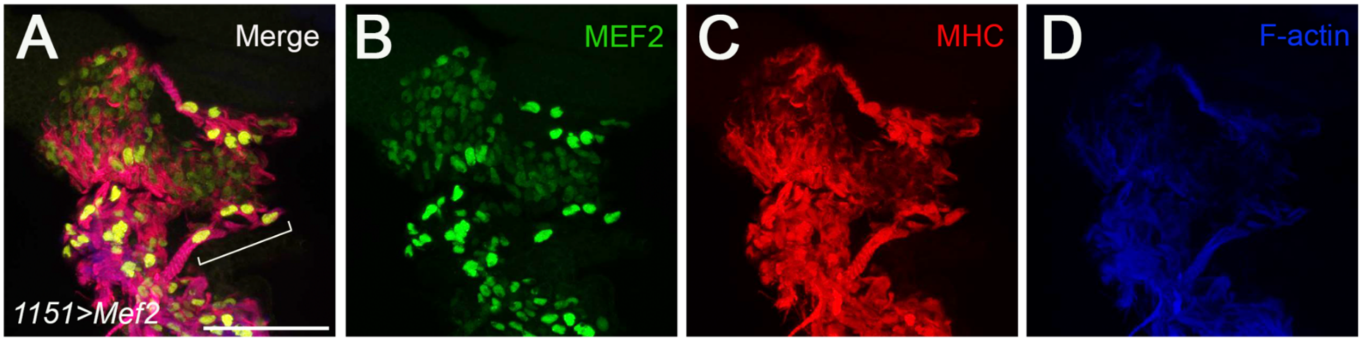

Figure S13. Phenotypic analysis of MEF2-induced myogenesis in wing discs of white pre-pupa reveals multinucleation. (A-D) *1151>Mef2* discs of white pre-pupa upon *Mef2* over-expression stained against MEF2, MHC, and F-actin showed striated-like structures and multinucleation (white bracket). Experimental n=5. Scale bar: 50 μm.

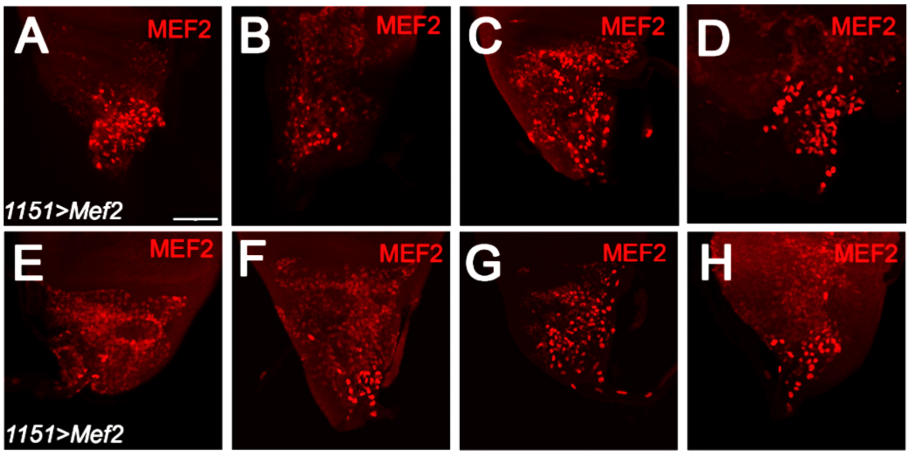

Figure S14. Immunofluorescence analysis of 1151>*Mef2* late third instar wing discs show elevated *Mef2* expression. MEF2 counterstain for (A) Bent (B) MHC (C) Unc-89 (D) Actn (E) Sls (F) Tm1 (G) TpnC and (H) Zasp52. Quantification can be found in Table 2. Scale bar: 50 μm.
